# Influence of different lubrication strategies on surface quality and geometric accuracy in incremental sheet forming of stainless steel

**DOI:** 10.1007/s00170-026-18677-3

**Published:** 2026-07-16

**Authors:** Aditya Vedanthu, Sabino Ayvar Soberanis, Chris M. Taylor, Pete Crawforth, David Curtis

**Affiliations:** The University of Sheffield Advanced Manufacturing Research Centre, Rotherham, UK

**Keywords:** Incremental sheet forming, Lubrication strategies, Friction indicator, Surface roughness, Geometric accuracy, Supercritical scCO₂, Minimum quantity lubrication

## Abstract

Incremental Sheet Forming (ISF) has gained significant attention as a flexible and cost-effective manufacturing method for producing low to medium volume and customised sheet metal components. However, its industrial uptake remains limited by gaps in knowledge related to surface quality and geometric accuracy, which are strongly governed by tribological conditions at the tool-sheet interface. This study experimentally compares five distinct lubrication strategies – Hydro slide (HG32) oil, flood emulsion coolant, minimum quantity lubrication (MQL) using soybean oil, supercritical CO₂ (scCO₂), and hybrid scCO₂ + MQL during 25 ISF trials of 1 mm thick stainless-steel grade 304 sheets formed into an 84 mm × 84 mm square pyramid geometry. Process performance was evaluated using vertical forming forces, a friction indicator derived from tangential (in-plane) and vertical force measurements, surface roughness parameters (Ra and Rz) measured using the Alicona optical topography functionality, and geometric accuracy using full field 3D scanning. The results showed that HG32 consistently produced the lowest vertical forming forces but resulted in the poorest surface quality, characterised by pronounced surface smearing, higher friction indicator values, and increased Ra and Rz roughness parameters. The higher friction indicator values suggest a greater contribution of tangential forces relative to vertical forces, suggesting increased tool-sheet friction and reduced lubrication effectiveness. In contrast, MQL achieved the best overall surface quality, a lower friction indicator value suggesting more favourable tool-sheet sliding conditions and improved lubrication effectiveness and the most stable geometric performance, despite generating higher forming forces than HG32. Full field deviation mapping showed that geometric deviations were concentrated along diagonal regions for all lubrication strategies. Overall, the findings provide a detailed process quality dataset and clear guidance on selecting lubrication strategies to balance force demand, surface quality, and dimensional accuracy in ISF of hard to form stainless-steel parts.

## Introduction

Incremental Sheet Forming (ISF) has emerged as a flexible manufacturing process capable of producing complex sheet metal components without the need for dedicated forming dies, in contrast to conventional sheet metal forming process such as deep drawing or stamping. ISF utilises a generic hemispherical tool that incrementally deforms a clamped sheet along a predefined toolpath, typically executed on Computer Numerical Control (CNC) machining systems. This incremental deformation mechanism allows complex geometries to be formed without part specific tooling, significantly reducing tooling cost and manufacturing lead time for low volume production and rapid prototyping applications [1]. Consequently, ISF has attracted considerable interest in industrial sectors such as aerospace, automotive and biomedical engineering where customised components and small production batches are common [2].

The deformation mechanism in ISF differs fundamentally from conventional sheet forming process. Instead of imposing deformation globally through rigid die sets, the forming tool progressively induces localised plastic deformation along the programmed toolpath. This localised deformation mechanism provides greater geometric flexibility and enables the formation of complex shapes that would be difficult or uneconomical to manufacture using conventional forming operations [3]. Furthermore, because the final component geometry can be modified simply by altering the programmed toolpath, the process is well suited to flexible manufacturing environments and modern digitally controlled production systems.

In addition to metallic materials, incremental forming has also been successfully applied to polymeric sheets and composite materials. Recent studies have demonstrated that polymers such as polycarbonate can be incrementally formed using similar deformation mechanism to metallic sheets, thereby expanding the range of materials that can be processed using ISF [4]. Research into the deformation behaviour and formability of ISF processes has also shown that process parameters such as step size, feed rate, and toolpath strategy strongly influence forming limits and process stability [5]. Despite these advantages, several technical challenges continue to limit the widespread industrial implementation of ISF. Compared with conventional die forming, incremental forming can, depending on process parameters and material properties, exhibit relatively higher forming forces, reduced dimensional accuracy, and poorer surface finish [6]. In addition, the absence of full die support during deformation can lead to non-uniform thickness distribution and spring back in the final component geometry. Improving process stability, surface integrity and dimensional accuracy therefore remain an important research objective in ISF development.

Several variants of the ISF process have been proposed in order to address these limitations. The most widely studied configuration is Single Point Incremental Forming (SPIF), in which a single forming tool progressively deforms the sheet while the sheet edges remain clamped, and the opposite side is unsupported [7]. To improve support conditions and dimensional control, Two Point Incremental Forming (TPIF) introduces a supporting die on the opposite side of the sheet [8]. More advanced configurations such as Double-Sided Incremental Forming (DSIF) utilises two synchronised tools acting on both sides of the sheet to improve geometric accuracy while retaining the flexibility of incremental forming [9]. In addition, hybrid and energy-assisted incremental forming process, including thermally assisted forming and electrically assisted forming techniques, have been investigated to improve formability and reduce forming forces when processing difficult to form materials [10].

Across all ISF variants, tribological behaviour at the tool-sheet interface plays a critical role in determining process performance. During incremental forming, the forming tool continuously slides across the sheet surface under high contact pressure conditions, generating friction and wear that influences forming forces, tool life and the surface quality of the formed component [11]. Inadequate lubrication may lead to adhesion, galling, and severe tool marks on the sheet surface, particularly when processing high strength alloys [12]. Furthermore, lubrication conditions interact with process parameters such as feed rate, toolpath strategy, and forming speed, thereby affecting forming forces and dimensional accuracy during ISF operations.

Consequently, significant research attention has been directed towards improving lubrication strategies for incremental sheet forming. Studies have shown that appropriate lubrication can significantly reduce friction and improve surface finish during SPIF operations [13]. Minimum Quantity Lubrication (MQL) has been investigated as a sustainable lubrication approach capable of improving tribological performance while significantly reducing lubricant consumption compared with convention flood cooling systems [14, 15].

Alternative lubrication approaches have also been explored. Graphite grease-based lubricants have demonstrated improved lubricant retention at the tool-sheet interface and reduced forming forces during incremental forming operations [16]. Vegetable oil-based nano lubricants containing SiO₂ nanoparticles have also been shown to improve boundary lubrication behaviour and enhance wear resistance during SPIF processes [17]. Additionally, specialised lubrications delivery systems such as roller-ball lubrication tools have been proposed to maintain continuous lubrication during heat-assisted incremental forming processes [18].

From a tribological perspective, lubrication performance is strongly influenced by lubricant chemistry and boundary film formation mechanism. Classical tribological literature describes how boundary lubrication film form through adsorption of polar molecules on metallic surfaces under high contact pressures [19]. In industrial forming processes, oil-in-water emulsions are commonly used due to their combined cooling and lubrication capability, achieved through the presence of base oils, surfactants and corrosion inhibitors [20]. Friction modelling studies have also demonstrated that lubrication conditions strongly influence friction behaviour and material flow during sheet metal forming processes [21]. Vegetable oil-based lubricants have attracted increasing attention as environmentally sustainable alternatives to conventional mineral oils due to their biodegradability and favourable tribological properties. The polar ester functional groups present in vegetable oils promote adsorption onto metallic surfaces, facilitating the formation of stable boundary lubrication films under severe contact conditions [22].

Despite the wide range of lubrication strategies investigated in the literature, emerging technologies such as supercritical carbon dioxide (scCO₂) and hybrid scCO₂ + MQL systems have not yet systematically studied for incremental sheet forming. scCO₂ possesses unique thermophysical properties including high diffusivity and low viscosity, which may enable improved penetration and cooling performance at the tool-sheet interface. These characteristics have the potential to influence friction behaviour, forming forces, surface quality, and dimensional accuracy during incremental forming operations. Therefore, the present study investigates the influence of five fundamentally different lubrication strategies on incremental sheet forming of stainless-steel sheets. The lubrication strategies evaluated include Hydro-slide oil (HG32), conventional flood emulsion coolant, Minimum Quantity Lubrication using soybean oil, supercritical carbon dioxide (scCO₂), and a hybrid scCO₂ + MQL system. A total of 25 forming trials were conducted on 1 mm thick stainless-steel grade 304 sheets to produce an 84 mm x 84 mm square pyramid geometry. The influence of lubrication strategy was evaluated in terms of vertical and tangential forming force, friction indicator (µ*) quantifying the tribological behaviour and contact resistance between the forming tool and the clamped sheet, surface roughness parameters (Ra and Rz), and geometric accuracy measured using full-field three-dimensional scanning.

## Experimental equipment and process

### Machine tool, forming test rig, and forming tool

**Fig. 1:**
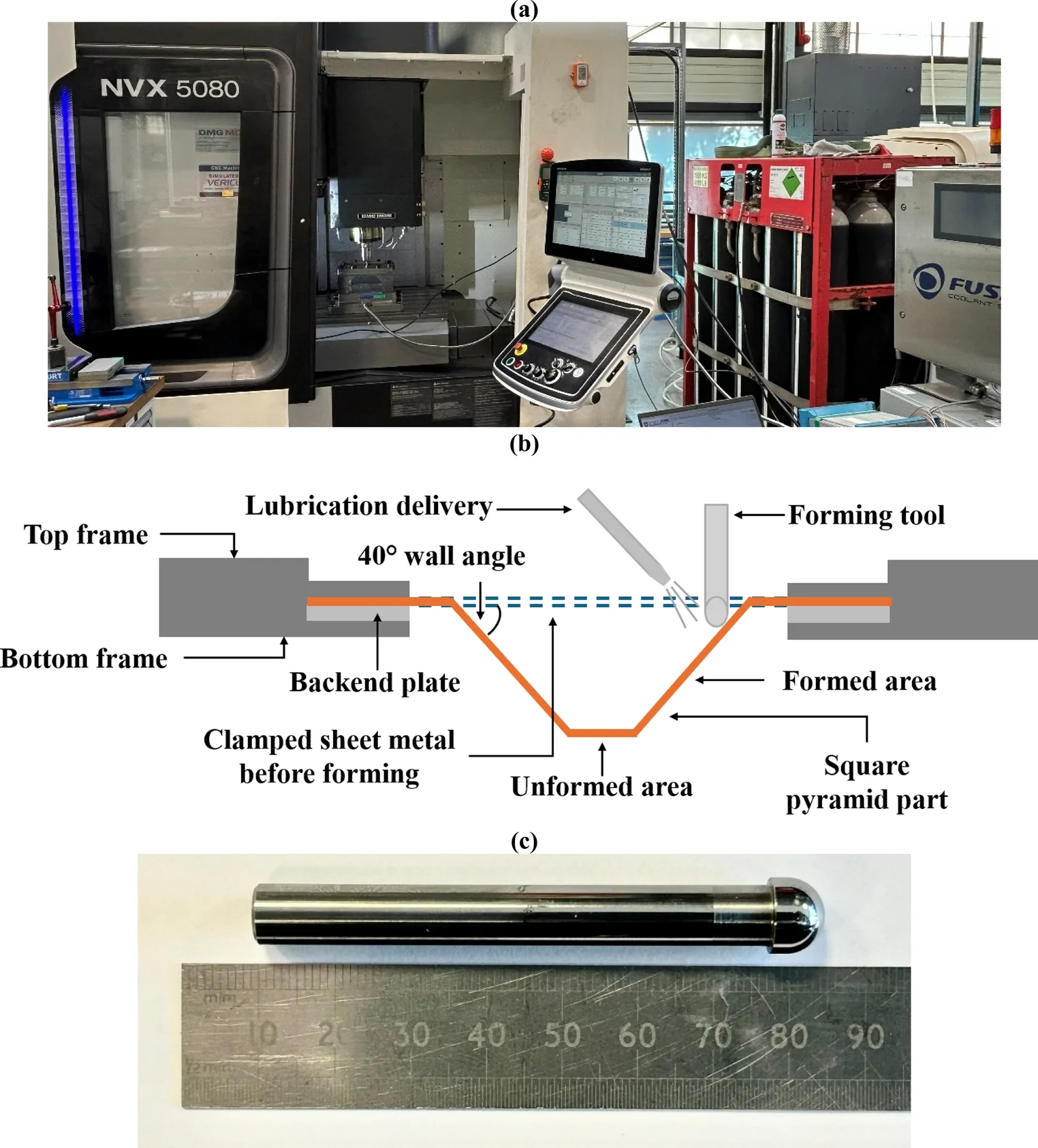
Experimental setup for incremental sheet forming: (**a**) DMG MORI NVX 5080 machining centre, (**b**) rigid steel forming rig (dynamometer-matched footprint), (**c**) WC hemispherical tool T0 (Ø10 mm)

The experimental program was designed to systematically evaluate the effects of five lubrication methods on the forming force, surface quality, and geometric accuracy of stainless-steel sheets under controlled laboratory conditions. All forming trials were conducted on a DMG MORI NVX 5080 (Fig. 1a) high precision vertical CNC machining centre, selected for its combination of rigid structure, high resolution control, and thermal stability, which are essential for consistent and repeatable incremental forming experiments. The NVX 5080 features axis travels of approximately 800 mm (X) x 540 mm (Y) x 510 mm (Z) with a working table capable of supporting up to 1000 kg, providing sufficient workspace for the forming rig and positioning auxiliary instrumentation. Its spindle system supports high speed rotation up to 15,000 RPM, and its advanced CNC interpolation ensures smooth execution of complex helical toolpaths while mitigating undesired vibrations in the machine structure. The platform’s precision, rigidity, and repeatability were critical to accurately capturing the effects of lubrication on forming forces, surface morphology, and dimensional deviations while a fully instrumented measurement chain recorded the process response throughout each trial.

The test rig was fabricated from structural steel to provide high rigidity and mechanical strength, ensuring minimal deflection under forming loads. Its footprint was carefully designed to match the Kistler 9255 C dynamometer dimensions (256 mm × 256 mm), promoting uniform load transfer and minimising bending moment artefacts that could distort the measured force signals. This configuration allowed the recorded process forces to be attributed to the incremental sheet forming operation, with negligible contribution from secondary structural compliance. A rigid, stack-type clamping fixture was developed to provide repeatable and high-stiffness boundary conditions during ISF. The fixture comprises of a steel bottom frame rigidly mounted to the test rig, onto which a steel back end (backing) plate is seated. The sheet metal is positioned directly on the back-end plate, and a top steel frame is subsequently installed to form a sandwich configuration (bottom frame – back-end plate – sheet metal – top frame), as seen in Fig. 1b. The assembly is secured using eight M8 bolts located around the periphery and tightened to a controlled torque of 15 Nm. Bolt tightening and loosening are performed using a star (cross) sequence to promote uniform clamping pressure and minimise local preload variation. For all experimental trials, the same bolt sequence and torque procedure was strictly applied during both clamping and unclamping to ensure consistent boundary conditions, reduce variability in sheet constraint and spring back, and maintain experimental repeatability and robustness.

The forming tool designated ‘T0’, with a nominal diameter of 10 mm, was supplied by Technicut Ltd. The tool had a conventional hemispherical ended design with an overall length of 73 mm (Fig. 1c) and was manufactured from uncoated tungsten carbide (WC), providing high dimensional accuracy and good wear resistance over the duration of the experiments. To ensure experimental consistency and isolate the influence of lubrication conditions, a new T0 tool was used for each lubrication strategy. This approach eliminated the progressive wear effects associated with repeated use of the tool and prevented inconsistency between lubrication conditions. Consequently, any observed variations in forming forces, surface quality or geometric response is solely attributed to the lubrication method rather than to changes in the tool condition.

### Part geometry, toolpath strategy, and forming parameters

**Fig. 2:**
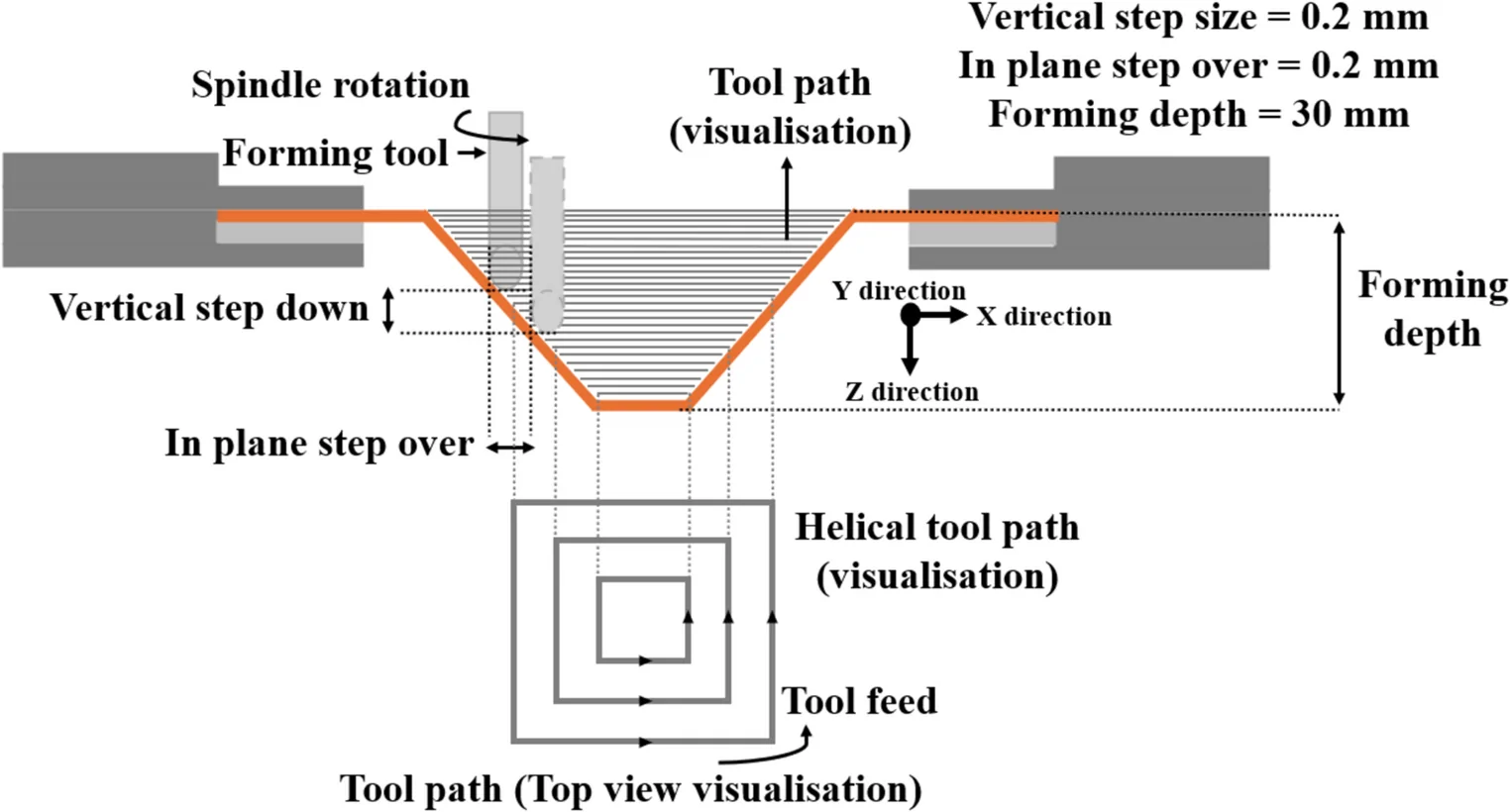
Toolpath - helical toolpath visualisation

Forming experiments were conducted using commercially relevant stainless-steel Grade 304 rolled sheets, all with a nominal thickness of 1 mm. Stainless-steel was specifically chosen due to its high strength and relatively low ductility, making it a challenging material to form and providing an opportunity to rigorously assess the effects of different lubrication strategies on hard-to-form alloys. The target component geometry was a square pyramid selected to facilitate straightforward benchmarking measuring 84 mm × 84 mm × 30 mm. A continuous helical milling toolpath strategy (Area Mill) was generated using Siemens NX software, as it closely follows the contours of the target geometry, ensuring smooth and uniform engagement between the forming tool and the sheet. The Area mill approach is particularly suited for ISF because it provides consistent step-over and incremental depth control (Fig. 2), avoids sudden direction changes and acceleration discontinuities, and maintains a continuous tool engagement condition, thereby reducing localised strain concentrations and promoting stable and uniform material flow along the walls. The chosen geometry provides a consistent benchmark for assessing the influence of different lubrication strategies under controlled process conditions. Additionally, a 40° wall angle ensured repeatability across trials and facilitated direct comparison of results, while remaining within the achievable forming limits for this material and avoiding premature fracture or excessive thinning during forming.

Two process parameter sets (Table 1) were evaluated to investigate the influence of lubrication strategies on forming forces, friction indication, surface roughness, and geometric accuracy. A vertical step size of 0.2 mm was selected to enable progressive and stable deformation of the stainless-steel sheet during forming, while the in-plane and step-over distance was kept constant at 0.2 mm for all trials. The feed rate and spindle speed were varied between two parameter sets, as these parameters have a dominant influence on contact conditions and material flow during ISF.

**Table 1:** Process parameters used for incremental sheet forming experiments

Parameter	Set 1 (F500, S0)	Set 2 (F750, S50)
Feed rate (mm/min)	500	750
Spindle speed (RPM)	0	50
Vertical step size (mm)	**0.2**	**0.2**
In plane step over (mm)	**0.2**	**0.2**

### Lubrication types

Five fundamentally different lubrication strategies were investigated to evaluate their influence on forming force, friction indication, surface roughness, and geometric accuracy. The selected lubrication methods span conventional lubrication oils, flood emulsion coolant, minimum quantity lubrication (MQL) with soybean oil, supercritical scCO₂ cooling, and a hybrid scCO₂ + MQL approach.

#### HG32 hydro slide oil

**Fig. 3:**
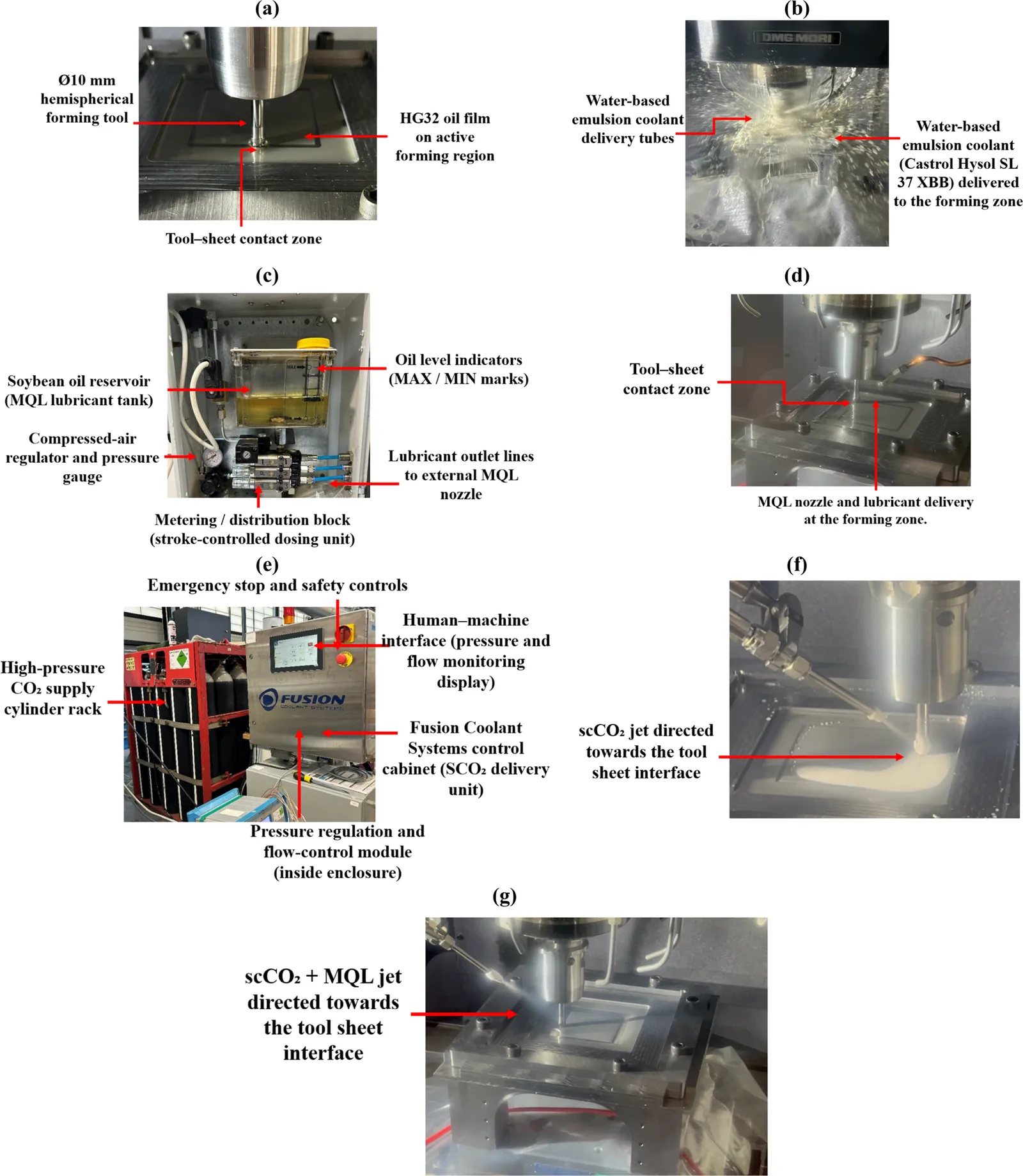
Lubrication and cooling systems used in ISF trials: (**a**) HG32 hydro-slide oil on the forming zone, (**b**) Castrol Hysol SL 37 XBB emulsion coolant delivery, (**c**) Allube Ltd MQL unit, (**d**) MQL nozzle position at the tool-sheet interface, (**e**) Fusion Coolant Systems unit for scCO₂ delivery, (**f**) scCO₂ nozzle in operation, (**g**) combined scCO₂ + MQL delivery at the tool-sheet interface

HG32 hydro slide oil was employed to promote stable boundary film formation under conditions of high contact pressure and limited hydrodynamic separation, typically enhanced using anti-wear and friction modifying additives. In this study, HG32 was applied manually applied with a brush to the sheet surface prior to forming (Fig. 3a) to ensure uniform coverage of the contact zone. The lubricant layer was replenished three times at regular intervals to prevent debris accumulation and material adhesion at the tool sheet interface, thereby minimising galling, tool wear, and surface degradation. This lubrication approach was adopted as a conventional baseline against which the performance of alternative and advanced lubrication strategies could be systematically evaluated.

#### Castrol hysol SL 37 XBB coolant

Emulsion lubrication was implemented using the Intelligent Fluid Delivery & Recovery (IFDR) system integrated with the DMG MORI NVX 5080 machining centre. The system delivers a nominal 20 bar pressure oil in water emulsion (Castrol Hysol SL 37 XBB) through multiple adjustable nozzles directed toward the tool sheet interface (Fig. 3b) producing a fully flooded contact condition throughout the forming operation. Such emulsified coolants are widely used in forming applications due to their combined cooling and lubrication capability, achieved through the presence of base oils, surfactants to enhance wetting behaviour, and corrosion inhibitors to protect both the delivery equipment’s and machine tool’s components. During ISF, the continuous coolant flow promotes heat dissipation, reduces friction at the tool-sheet interface, and assists in the removal of wear debris or microscopic material fragments generated during sliding contact. This debris flushing action is particularly important in preventing adhesion, galling, and surface damage that may otherwise arise under sustained contact conditions.

#### MQL (soybean oil)

MQL was implemented using a system supplied by Allube Ltd (Fig. 3c), delivering undiluted soybean oil directly to the forming zone. Soybean oil was selected due to its biodegradability, low toxicity, and favourable tribological characteristics, making it a sustainable alternative to conventional mineral based lubricants. Chemically, soybean oil is a triglyceride composed predominantly of long chain unsaturated fatty acids, including oleic, linoleic, and linolenic acids. These molecules contain polar ester functional groups that promote strong adsorption onto metallic surfaces, facilitating the formation of stable boundary lubrication films. The affinity of vegetable oil-based lubricants to metal interfaces, arising from their molecular polarity, has been shown to enhance boundary lubricity and frictional stability when applied in minimal quantities, making soybean oil particularly suitable for MQL applications. The MQL nozzle was positioned such that the lubricant outlet was located within 30 mm of the tool sheet interface and oriented at an angle of 45° relative to the sheet surface, ensuring direct impingement on the contact zone (Fig. 3d). Careful nozzle placement enabled effective lubricant delivery while avoiding interference with the forming rig. The system was operated at a discharge volume of 16.03 mm³ per cycle with 66 strokes per minute, corresponding to a volumetric flow rate of 1058 mm³/min (or 63.48 mL/h). This configuration provides targeted lubrication with minimal fluid consumption. The pressurised air stream accompanying the lubricant delivery further assisted in the removal of wear debris or microscopic material fragments generated at the tool sheet interface, thereby supporting continuous lubrication, reducing the likelihood of adhesion or galling. In terms of adoptability, the economic and environmental burden associated with ownership of MQL systems is very reasonable. As an indication, systems cost £100 to purchase and at the flowrate quoted above, the associated lubricant consumption cost is around £1 per hour. Electrical power consumption is in the order of 8 W.

#### scCO₂

Supercritical carbon dioxide (scCO₂) lubrication was implemented using the Fusion Coolant Systems unit, as shown in Fig. 3e. The system is designed to deliver pressurised CO₂ based lubricant media through precision micro-nozzles, enabling controlled jetting directly to the tool workpiece interface. The compact stainless-steel enclosure houses the pressure regulation, flow control, and safety systems required for stable scCO₂ operation during the forming process. The Fusion system was operated using an exit nozzle diameter of 0.25 mm, selected to promote high jet velocity and focused delivery at the forming zone (Fig. 3f). The system was configured with the following operating parameters: the CO₂ supplied at an average temperature of 41.9 °C to 43.1 °C and pressure of 98–99 bar. The overall CO₂ mass flow rate was 7.74 kg/h. This flow rate configuration was intentionally selected to closely match the MQL lubrication rate used in the MQL trials, ensuring a consistent basis for comparison between lubrication strategies. The scCO₂ jet was directed towards the tool-sheet interface to provide localised cooling while minimising overall fluid consumption.

#### scCO₂ + MQL

A hybrid lubrication strategy combining scCO₂ and MQL was also evaluated using the same Fusion Coolant Systems unit. The system is capable of delivering both scCO₂ and liquid MQL through a common delivery architecture (Fig. 3g), allowing simultaneous application at the forming interface. In this configuration, soybean oil was used as the liquid lubricant, consistent with the standalone MQL trials. The fusion system was operated under the same discharge conditions as the scCO₂ only configuration. CO₂ was supplied at an average temperature of 41.9 °C to 43.1 °C and pressure of 98–99 bar. For the MQL system, the operating parameters were 33.7 mm³ per cycle, 37 strokes per minute, and a total flow rate of 1246.9 mm³/min (74.8 mL/h). This ensured that the hybrid scCO₂ + MQL condition closely matched to the standalone MQL flow rate, allowing direct comparison of lubrication effectiveness. For both the scCO₂-only delivery and the scCO₂ + MQL delivery, a nozzle diameter of 0.25 mm was used producing a focused jet aimed at the tool-sheet contact region. The combined approach was intended to exploit the penetration and cooling capability of scCO₂ together with the lubricity of soybean oil, while maintaining low lubricant consumption, controlled delivery and minimal surface oil residue.

## Instrumentation details and measurement methodology

A set of complementary measurement systems were used to characterise the incremental sheet forming process under different lubrication conditions. The instrumentation was selected to measure forming forces, surface quality, and geometric accuracy, which are the key indicators of process performance in ISF. Force data were acquired during forming, while surface and geometric measurements were conducted post process using non-contact metrology systems.

### 9255 C Kistler dynamometer

Forming forces were measured using a Kistler 9255 C piezoelectric dynamometer (Fig. 1a), capable of simultaneously recording force components in the X, Y, and Z cartesian directions. The dynamometer was mounted directly beneath the forming rig, allowing accurate measurement of the forces transmitted during the ISF process. The device has a 256 mm × 256 mm footprint, which was matched by the rig design to ensure uniform load transfer and to minimise bending effects or parasitic structural influences. The dynamometer signals captured at a frequency of 50,000 Hz were conditioned using a Kistler charge amplifier and recorded through the in-house developed MATLAB based data acquisition system, ensuring repeatable and reliable force measurements for all experimental trials.

The analysis presented in this study focuses on two key force components. First, the vertical force component ($\:{F}_{z}$), which acts normal to the sheet surface and represents the primary forming load in ISF. Second, the tangential force, ($\:{F}_{t}$), calculated as $\:{F}_{t}=\sqrt{{F}_{x}^{2}+{F}_{y}^{2}}$ which represents the resultant in-plane force acting parallel to the sheet surface. The tangential force characterises the lateral interaction between the forming tool and the sheet as the tool traverses the programmed toolpath and is used to determine the friction indicator ($\:{\mu\:}^{*}$), defined as the ratio of tangential force to vertical force. A similar force ratio-based friction indicator approach was adopted by Körner and Ou [23] to investigate frictional behaviour during incremental sheet forming of titanium sheets. Equation 1 presents the formulation used to calculate ($\:{\mu\:}^{*}$).

1 $$\:{\mu\:}^{*}=\frac{{F}_{\mathrm{t}}}{{F}_{z}}=\frac{\sqrt{{F}_{x}^{2}+{F}_{y}^{2}}}{{F}_{z}}$$

The friction indicator reflects the combined influence of tool-sheet frictional interaction and the vertical forming load required to plastically deform the material. Higher ($\:{\mu\:}^{*}$) value indicate a greater contribution of tangential force relative to the vertical force, suggesting increased resistance to tool sliding and less favourable tribological conditions at the tool-sheet interface. Conversely, lower ($\:{\mu\:}^{*}$) values indicate reduced resistance to tool sliding, reflecting more favourable contact conditions and improved lubrication effectiveness during the forming process.

### Alicona optical micro-CMM

Surface quality of the formed parts was characterised using an Alicona optical micro-coordinate measuring machine (Micro-CMM) based on focus variation technology. This non-contact measurement system enables high-resolution three-dimensional surface reconstruction, making it particularly suitable for assessing ISF-induced surface features such as tool marks, waviness, and localised roughness variations. The Alicona system provides sub-micron level vertical resolution, and the surface roughness was quantified using the arithmetic mean roughness (Ra) and the maximum height of the profile (Rz), in accordance with ISO 21,920.

**Fig. 4:**
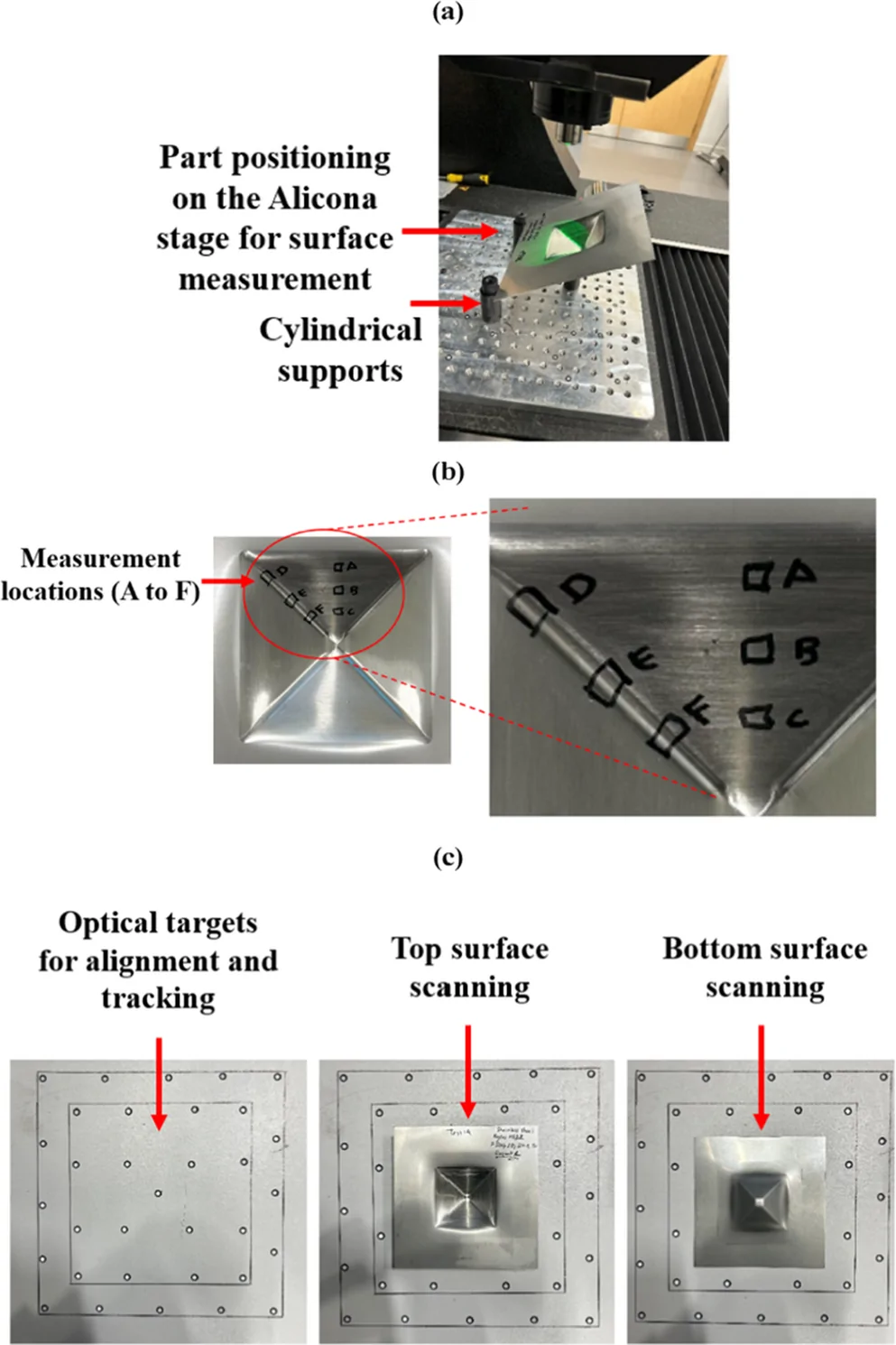
Metrology and scanning setup: (**a**) part positioning on Alicona stage; (**b**) surface measurement locations; (**c**) optical alignment targets and 3D scanning procedure

The formed part having a 40° wall angle was positioned at a 40° angle using three cylindrical supports as seen in Fig. 4a to allow for the surface to be accurately read by the machine. Six measurement regions, labelled A to F (Fig. 4b), were consistently evaluated on each formed component under all lubrication conditions to ensure direct comparability of surface measurements. Regions A to C were located along the inclined wall of the square pyramid to characterise surface quality on the primary forming surface, while Regions D to F were positioned along the diagonal edge to assess surface characteristics in geometrically complex regions, where the local deformation state differs from that experienced on the planar wall. The selected locations also represent different stages of the forming process. Regions A and D correspond to the initial phase of the forming, whereas Regions C and F were located near the final forming depth. All measurement points were equally spaced along the formed surface to ensure a balanced and unbiased assessment of surface evolution.

In addition to the conventional surface roughness parameters (Ra, and Rz), high resolution surface texture plots were generated at the distinct locations (A to F). Each texture plot represents a scan area of 0.095 mm in width and 1.71 mm in height, providing a detailed topography of the formed surface. The scans were performed at an estimated vertical resolution of 50 nm and a lateral resolution of 2.93 μm, ensuring precise capture of sub-micron surface features. Imaging parameters were carefully controlled, with an exposure time 2.45 ms, contrast setting of 0.43 ms, saturation and gain set to 1, and a magnification of 20.2208×. Illumination was provided by the SmartFlash Lightsource G2, utilizing both coaxial and ring lighting with full illumination to optimize the visualisation of micro geometry and surface anisotropy. The resulting texture plots not only visualise surface roughness features but also provide critical insights into lubrication-induced patterns, which are not fully captured by scalar roughness parameters alone.

### Creaform handheld 3D laser scanner

Full field geometric accuracy of the formed parts was evaluated using a Creaform handheld 3D laser scanner. The analysis quantified a geometric comparison between the formed part and its nominal design geometry, used to assess dimensional accuracy, shape fidelity, and the effects of lubrication methods. Following forming, the parts (both the top surface and bottom surface) were digitised using a Creaform Handheld 3D laser scanner, generating a high-density point cloud that captures the true as-formed surface geometry. The top part and the bottom part were then merged into one scan. This scan data was exported as an STL file and represents the experimental geometry of the part. To enable an accurate and meaningful comparison, the scanned geometry was aligned to the original CAD model within MATLAB using a robust multi-stage registration method. First, a coarse alignment was achieved using centroid matching and principal component analysis (PCA) to correct for gross positional and orientational difference between the two datasets. This was followed by an Iterative Closest Point (ICP) registration procedure, which refined the alignment by minimising the point-to-point distance between the scanned and nominal surfaces. This ensured that any remaining deviation was due to forming and material behaviour. Following alignment, a nearest neighbour search algorithm (kNN) was used to associate each point on the scanned surface with the closest corresponding point on the CAD model. The deviation at each location was then computed by projecting the vector difference between these paired points onto the surface normal of the nominal CAD geometry. This approach provides both the magnitude and direction of deviation, allowing the distinction between inward and outward dimensional errors. The resulting deviations were visualised using 2D and 3D contour maps, with a consistent colour scale denoting positive and negative variances in millimetres. These plots enabled identification of regional trends such as bulging or asymmetric deformation, features commonly associated with ISF due to localised strain, and material anisotropy.

An initial 3D optical scanner calibration was performed using the VX Elements scan module in Creaform. This calibration verifies accuracy by measuring a high precision reference plate and adjusting the scanner’s internal optical and electronic parameters to match known dimensions. The procedure compensates for environmental effects such as temperature fluctuation or minor mechanical vibrations, ensuring consistent, repeatable, and reliable 3D measurement data while minimising point cloud errors. A residual quality error of 0.011 mm was achieved, which is well within the Creaform specific accuracy requirement of 0.025 mm. Reflective targets were positioned around the part to provide a stable spatial reference (Fig. 4c) for the scanner enabling real time tracking of its 6-DOF position and accurate registration between successive frames. This is critical for smooth, low texture, or symmetric surfaces where geometry-based tracking alone is unreliable. Targets were arranged in a symmetrical distribution ensuring at least four targets always remain visible. Proper target placement reduces drift, improves alignment between viewpoints, and enhances overall dimensional accuracy and repeatability of the final point cloud and mesh. The upper surface of the part (Fig. 4c) was then scanned by gradually moving the scanner around the part while maintaining the standoff distance which is seen as a green light on the scanner. The part was subsequently flipped (Fig. 4c), and the opposite surface was scanned allowing both the top and bottom geometries to be captured. Next the two scans were aligned, merged and exported as an STL file to MATLAB for deviation analysis.

## Results and discussion

### Forming force

This section examines the influence of lubrication strategy on the evolution of vertical and tangential forming forces during ISF. While lower forming forces are generally desirable for improved process stability and reduced tool deflection, force magnitude alone does not fully define process performance. Certain lubrication strategies, particularly coolant and scCO₂ based approaches, may increase forming forces due to enhanced cooling and reduced localised thermal stabilisation of the material, yet still offer advantages in terms of surface quality. These interactions are discussed here from a force generation perspective, with surface quality and geometric conformance addressed in detail in subsequent sections.

**Fig. 5:**
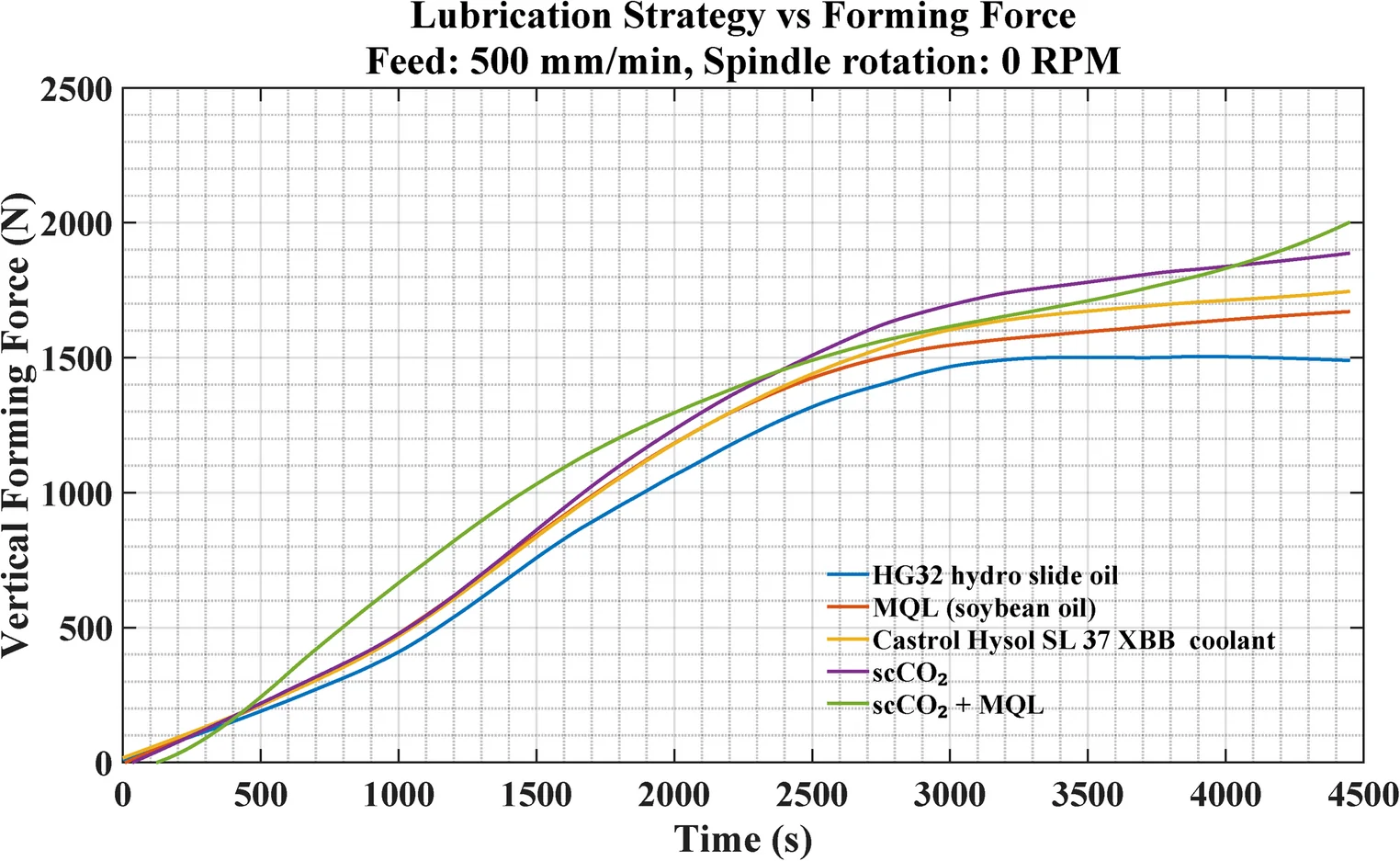
Vertical forming force response for different lubrication strategies for feed rate 500 mm/min and spindle speed 0 RPM

**Fig. 6:**
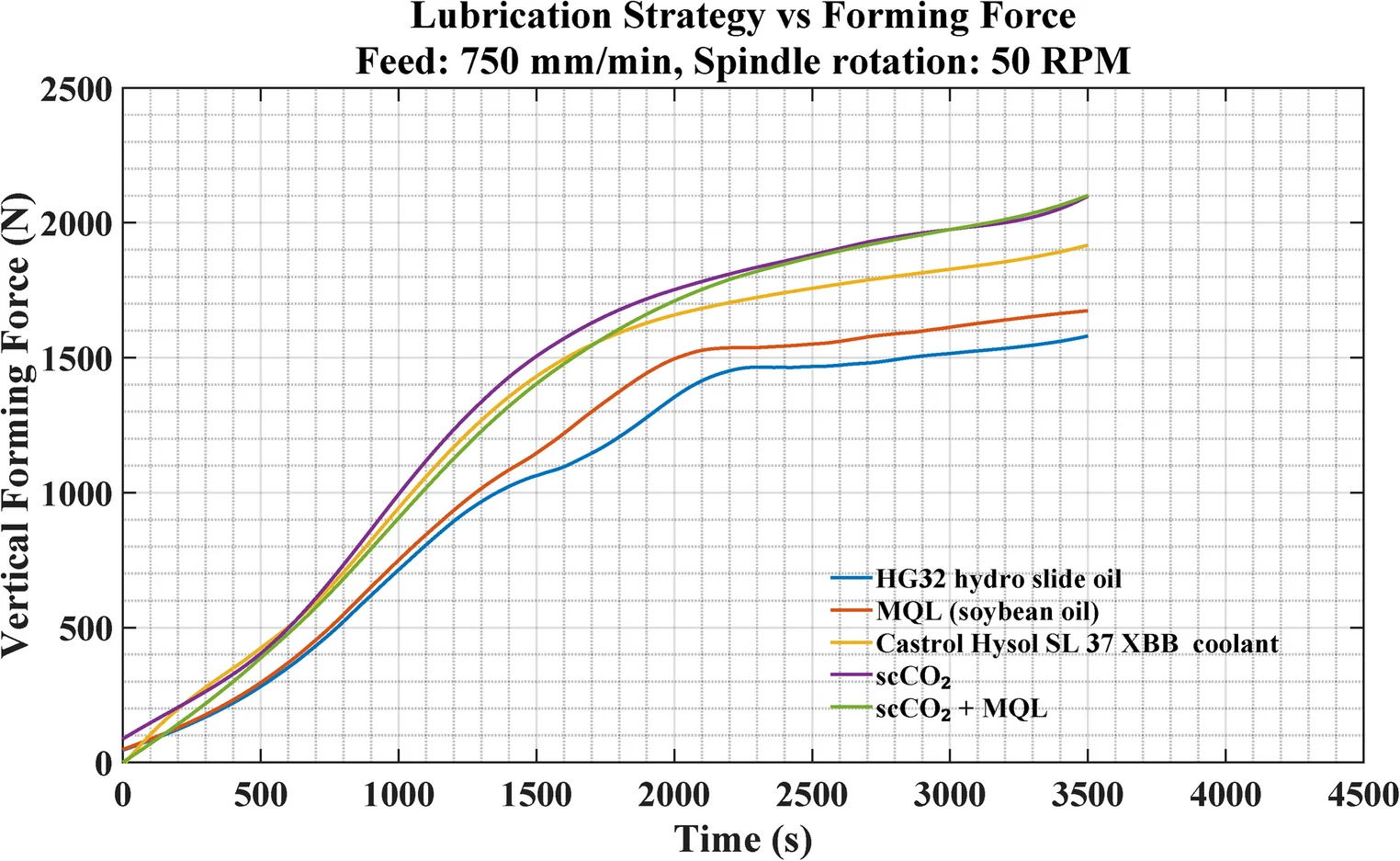
Vertical forming force response for different lubrication strategies for feed rate 750 mm/min and spindle speed 50 RPM

The forming force signals shown in Figs. 5 and 6 are based on the original measured data; however, for clarity of trend visualisation, the signals were post processed using a two-stage procedure. First, all datasets were interpolated onto a common time base to ensure consistent comparison between lubrication conditions, as the raw recordings exhibited slight variations in total acquisition duration. Subsequently, a Savitzky-Golay smoothing filter (third order polynomial with a wide frame length of 301) was applied to the data. This approach performs a local polynomial least squares approximation within a moving window rather than simple averaging, thereby preserving the intrinsic structure of the signal. In contrast to moving average filtering, the Savitzky-Golay method maintains both peak magnitude and local curvature, which is critical for retaining physically meaningful force evolution characteristics during forming tool-sheet metal interaction.

#### Forming force (F_z_) analysis (feed 500 mm/min, spindle speed 0 RPM)

Under a feed rate of 500 mm/min and zero spindle rotation, the vertical forming forces increase monotonically for all lubrication strategies as the forming depth progresses. Hydro slide (HG32) oil results in the lowest force levels throughout the forming cycle, reaching a peak force of approximately 1.5 kN. MQL and machine coolant generate intermediate force levels, while scCO₂ and scCO₂ + MQL exhibit the highest forces, with peak values exceeding 1.8 kN and 2.0 kN respectively (Fig. 5). A clear separation between lubrication strategies is observed under these conditions. Oil-based lubrication leads to lower force gradients and earlier force stabilisation; where coolant and scCO₂ based strategies show a steeper force increase, particularly at higher forming depths. The hybrid scCO₂ + MQL condition exhibits the most pronounced force escalation during the latter stages of forming. Under these conditions, coolant and scCO₂ primarily act as cooling medium rather than lubricants. Despite the higher force levels, these strategies are expected to reduce adhesive wear and surface damage, an effect that is examined in detail in Sect. 4.2.2.

#### Forming force (F_z_) analysis (feed 750 mm/min, spindle speed 50 RPM)

At a feed rate of 750 mm/min and a spindle speed of 50 RPM, HG32 remains the lowest force option, with a peak force of 1.6 kN. MQL and machine coolant generate moderate force levels, while scCO₂ and scCO₂ + MQL again produce the highest forces, approaching 2 kN towards the end of the forming cycle (Fig. 6). Although the ranking of lubrication strategies remains unchanged from above, the difference in force magnitude between them is reduced relative to the non-rotation condition. The introduction of spindle rotation results in smoother force evolution and a more gradual increase in forming force for all lubrication strategies. scCO₂ and scCO₂ + MQL show similar force trends over much of the forming cycle with divergence occurring at larger forming depths. scCO₂ and coolant-based approaches continue to generate higher forces indicating that enhanced cooling and limited heat retention still dominate the material response. While these higher forces may be detrimental from a tool load standpoint, they may contribute positively to surface quality by reducing galling, oxidation, and adhesive wear. These effects are quantitatively assessed in the subsequent surface quality analysis.

#### Friction Indicator analysis

**Fig. 7:**
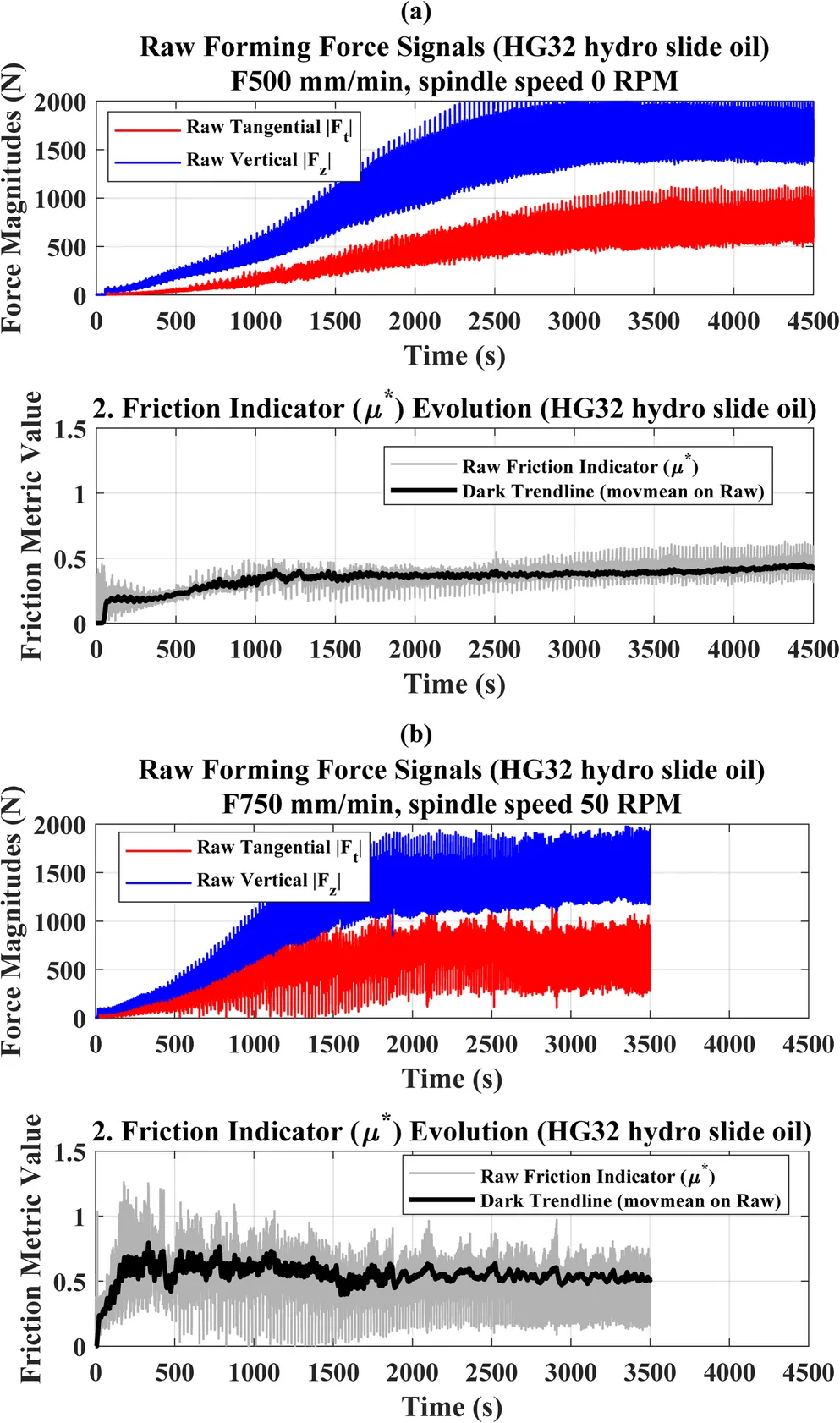
A typical force diagram and friction indicator evolution – HG32 hydro slide oil: (**a**) F500 mm/min, spindle speed 0 RPM; (**b**) F750 mm/min, spindle speed 50 RPM

Figure 7(a-b) depicts a representative dataset showcasing the transient evolution of the raw forming force signals alongside the derived friction indicator ($\:{\mu\:}^{*}$) using HG32 hydro slide oil strategy. For brevity and clear visualisation of the underlying signal processing methodology, only the HG32 strategy is explicitly plotted here, however, this exact analytical pipeline and force to friction relationship apply to all remaining lubrication strategies across both investigated process conditions (i.e., Feed: 500 mm/min, spindle speed: 0 RPM and Feed: 750 mm/min, spindle speed: 50 RPM).

Subplot 1 illustrates the continuous time series data for both the raw tangential and vertical forming force and subplot 2 illustrates the evolution of tribological conditions with time at the contact interface derived from the raw force inputs. The raw friction indicator values calculated directly as the instantaneous ratio of the tangential force to the vertical force are displayed in light grey. To isolate the underlying tribological trend, a Savitzky-Golay smoothing filter utilising a third order polynomial and a 301-point frame length was implemented to generate the robust trendline (solid black line).

The friction indicator, µ*, is a derived process metric used to characterise the relative contribution of in-plane (tangential) resistance to the vertical forming load during the ISF process. It is obtained by combining the two-horizontal force components ($\:{F}_{x}\:\&\:{F}_{y}$) recorded during tool-sheet interaction into a single resultant in-plane force ($\:{F}_{t})$, which is then expressed as a ratio against the simultaneously recorded vertical force ($\:{F}_{z})$. Physically, this ratio reflects how much of the tool’s vertical effort is being diverted into overcoming resistance to sliding at the tool-sheet contact, as opposed to the productive work of plastically deforming the sheet. Because (µ*) combines both the frictional interaction at the tool tip and the bulk vertical load required to form the material it should be interpreted as a process level indicator of tribological condition rather than a direct measured, decoupled coefficient of friction. On this basis, higher (µ*) value suggests a greater contribution of tangential force relative to vertical force, indicating increased tool-sheet friction and reduced lubrication effectiveness. In contrast, MQL achieved a lower friction indicator value suggesting more favourable tool-sheet sliding conditions and improved lubrication effectiveness.

**Fig. 8:**
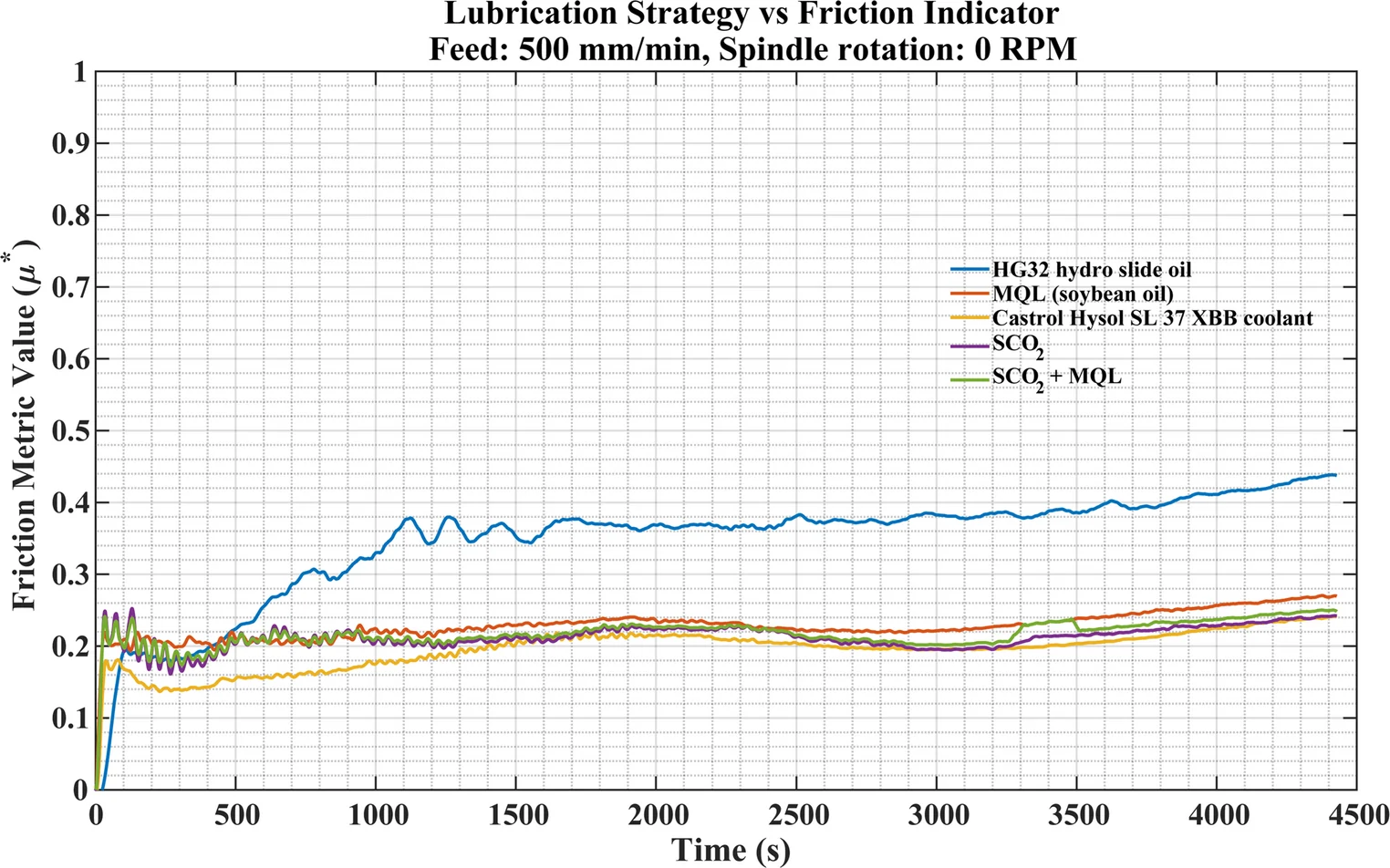
Friction indicator evolution – F500 mm/min, spindle speed 0 RPM

Figure 8 presents the Savitzky-Golay smoothed evolution of µ* over the trial duration for all five lubrication strategies at this condition (Feed: 500 mm/min, spindle speed: 0 RPM). HG32 shows a sustained, progressive increase, rising from an initial transient band of approximately 0.18–0.25 to a quasi-steady plateau of roughly 0.36–0.39 between 1500 s and 3500 s, before drifting further to approximately 0.43 by the end of the trial. This rise is consistent with the surface smearing and poor lubrication effectiveness associated with HG32, and is indicative of a less stable, higher friction contact regime. MQL, scCO₂, and the hybrid scCO₂ + MQL form a comparatively tight band across the bulk of the trial, settling between approximately 0.20 to 0.25, with MQL drifting gradually to ~ 0.27 by the end of the trial. Notably, the flood emulsion coolant records the lowest µ* vales of all five strategies through the early to mid-portion of the trial, before converging toward the MQL/scCO₂ band in the later half. This indicates that, at this specific feed and spindle condition, the flood coolant provides comparable sliding conditions than MQL for the majority of the test, although this should be considered alongside the surface roughness and geometric accuracy results presented in the sections ahead, since µ* alone does not establish which strategy produced the superior part outcome.

**Fig. 9:**
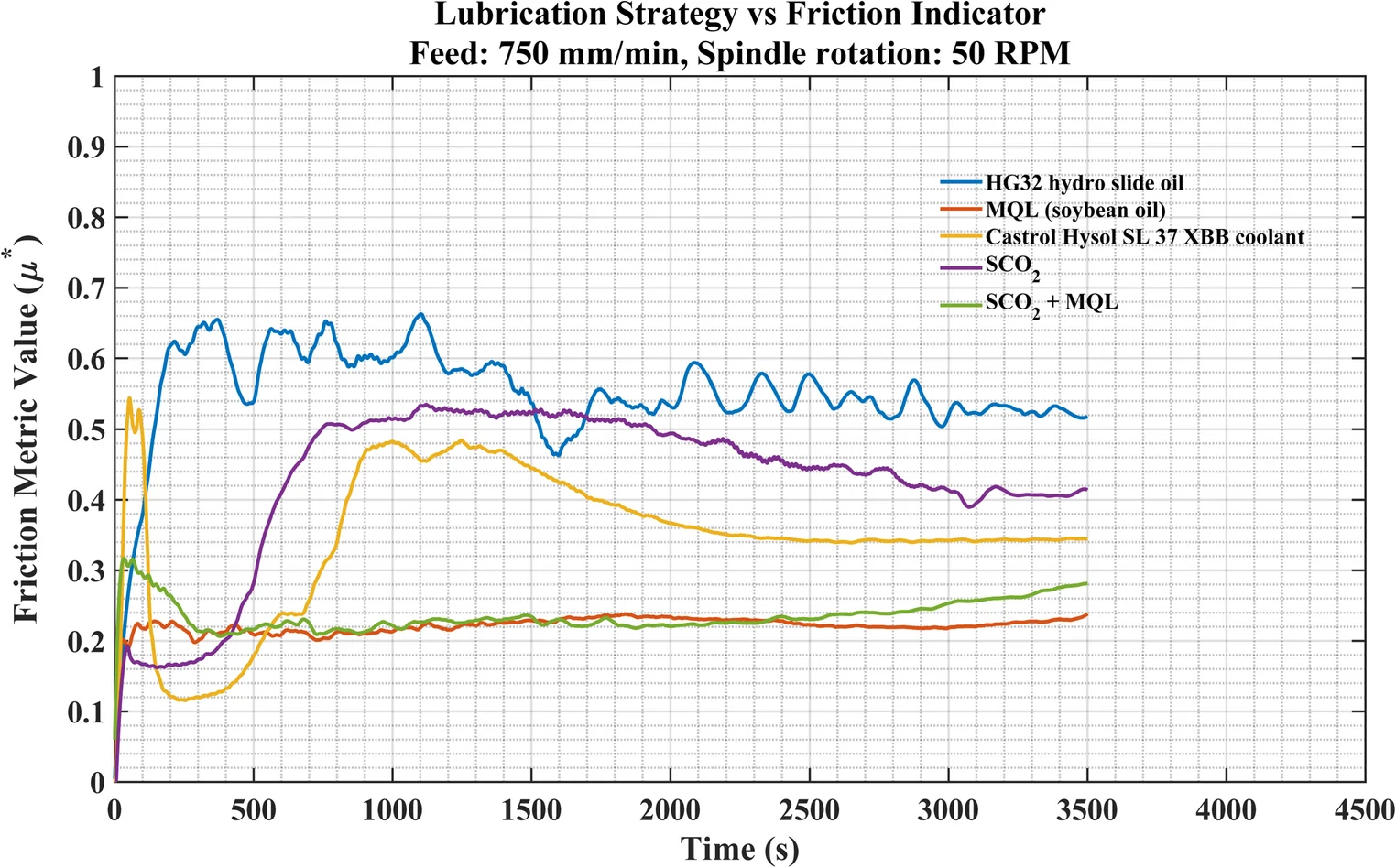
Friction indicator evolution – F750 mm/min, spindle speed 50RPM

At the increased feed rate of 750 mm/min with 50 RPM spindle rotation (Fig. 9), the relative ranking of the five lubrication strategies changes notably compared with the 500 mm/min, 0 RPM, condition, although the underlying relation between µ* and tribological performance is unchanged, higher µ* continues to indicate increased resistance to tool sliding and reduced lubrication effectiveness, while lower, more stable µ* indicates more favourable contact conditions. HG32 again produces the highest µ*, rising rapidly to approximately 0.76 within and oscillating between 0.50 and 0.70 thereafter, a substantially higher magnitude than recorded at the lower feed, non-rotation condition, suggesting that the combination of increased feed and spindle rotation amplifies the frictional penalty associated with HG32’s comparatively poor lubricating film. scCO₂ shows a delayed but pronounced rise, reaching a plateau of approximately 0.50–0.53 before declining to around 0.41 by the end of the trial The flood emulsion coolant exhibits a similarly delayed rise, plateauing at approximately 0.45–0.49 before falling and stabilising at approximately 0.34–0.34 for the remainder of the test. Both strategies therefore display transient mid trial elevations in µ* that are absent, or far less pronounced, at the 500 mm/min, 0 RPM condition. MQL and the hybrid scCO₂ + MQL remain the lowest and most stable of the five strategies throughout the trial, settling rapidly to approximately 0.21–0.24 and remaining within this band. MQL lubrication with a combination of higher feed rate of 750 mm/min with 50 RPM spindle rotation produced the most stable friction indicator evolution trend in comparison to the other lubrication techniques. This sustained, low amplitude response indicates that the MQL whether applied alone or in in combination with scCO₂ provides the most consistent tool-sheet sliding conditions of the five strategies.

### Surface quality

Surface quality is a critical attribute in incremental sheet forming, particularly for applications where functional performance and aesthetic requirements are important. In ISF, surface quality is strongly influenced by tribological conditions at the tool-sheet interface, where lubrication strategy governs friction, adhesion, and local thermal effects. While higher forming forces may be associated with using emulsion coolant or scCO₂ based options, they do not necessarily translate to poorer surface finish. This section therefore focuses on a quantitative assessment of surface roughness parameters, especially the arithmetic mean roughness (Ra) and the maximum height of the profile (Rz).

#### Alicona measurement repeatability

**Fig. 10:**
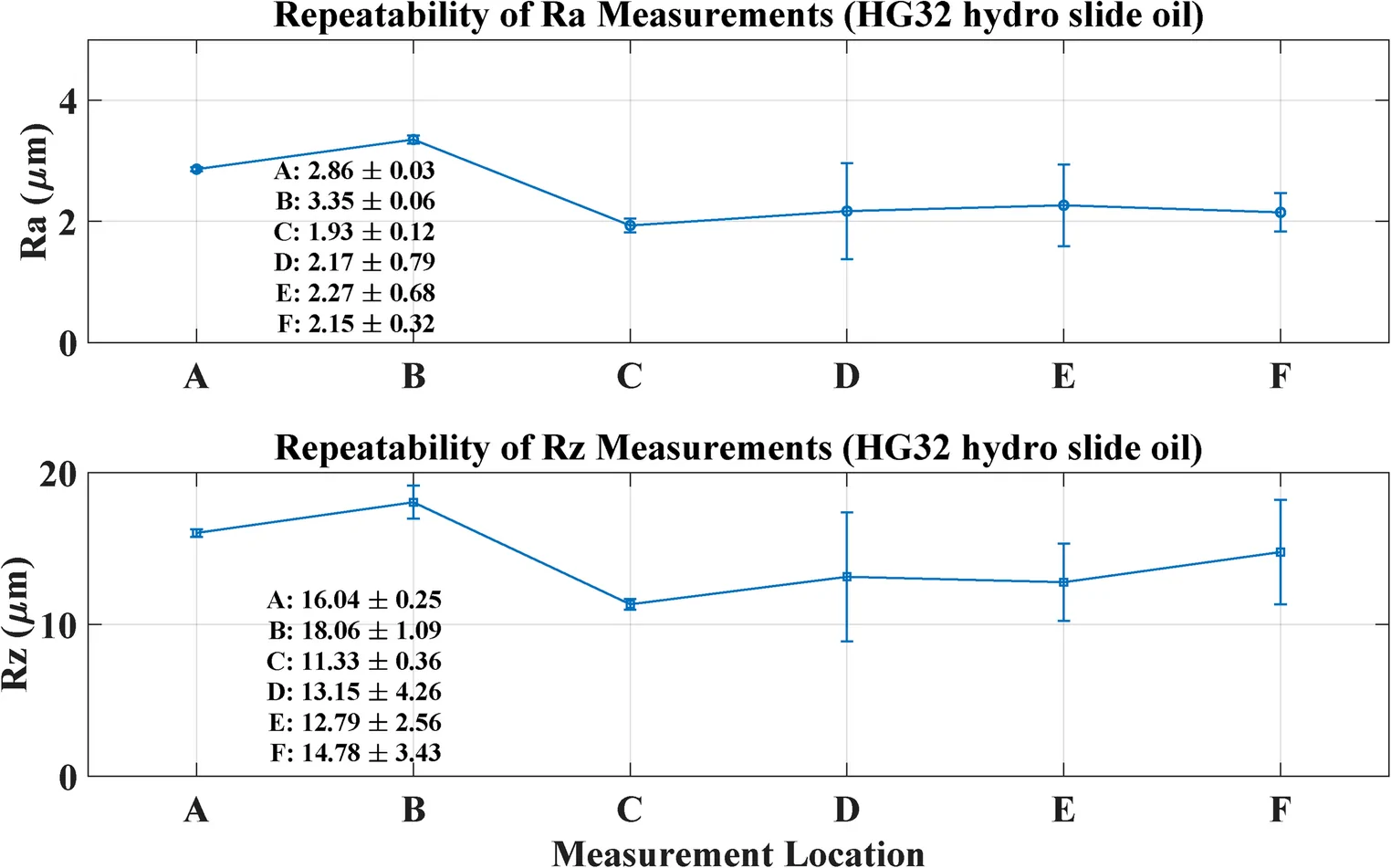
Ra and Rz repeatability error plot

To assess the repeatability and reliability of the surface roughness measurements, three repeated scans were conducted using HG32 hydro slide oil at the six predefined locations (A to F) on the formed component for both Ra and Rz. Measurement repeatability at each location was quantified by calculating the mean value and corresponding standard deviation from the repeated scans (Fig. 10). The variability in the measurements is illustrated using error bar plots, where the error bars represent standard deviation about the mean.

The results demonstrate a high level of measurement consistency for both Ra and Rz at locations A, B, and C, with only limited scatter observed at locations D, E, and F. The slightly increased variability at these locations is attributed to the more complex surface topography in the diagonal region of the square pyramid, as illustrated in Fig. 4b. Nevertheless, the measured standard deviation remains small relative to the corresponding mean values, indicating robust measurement stability. Overall, these results confirm that the Alicona optical measurement system provides adequate accuracy and repeatability for surface quality assessment in the present study.

#### Surface quality analysis-(feed rate 500 & 700 mm/min, spindle speed 0 & 50 RPM

**Fig. 11:**
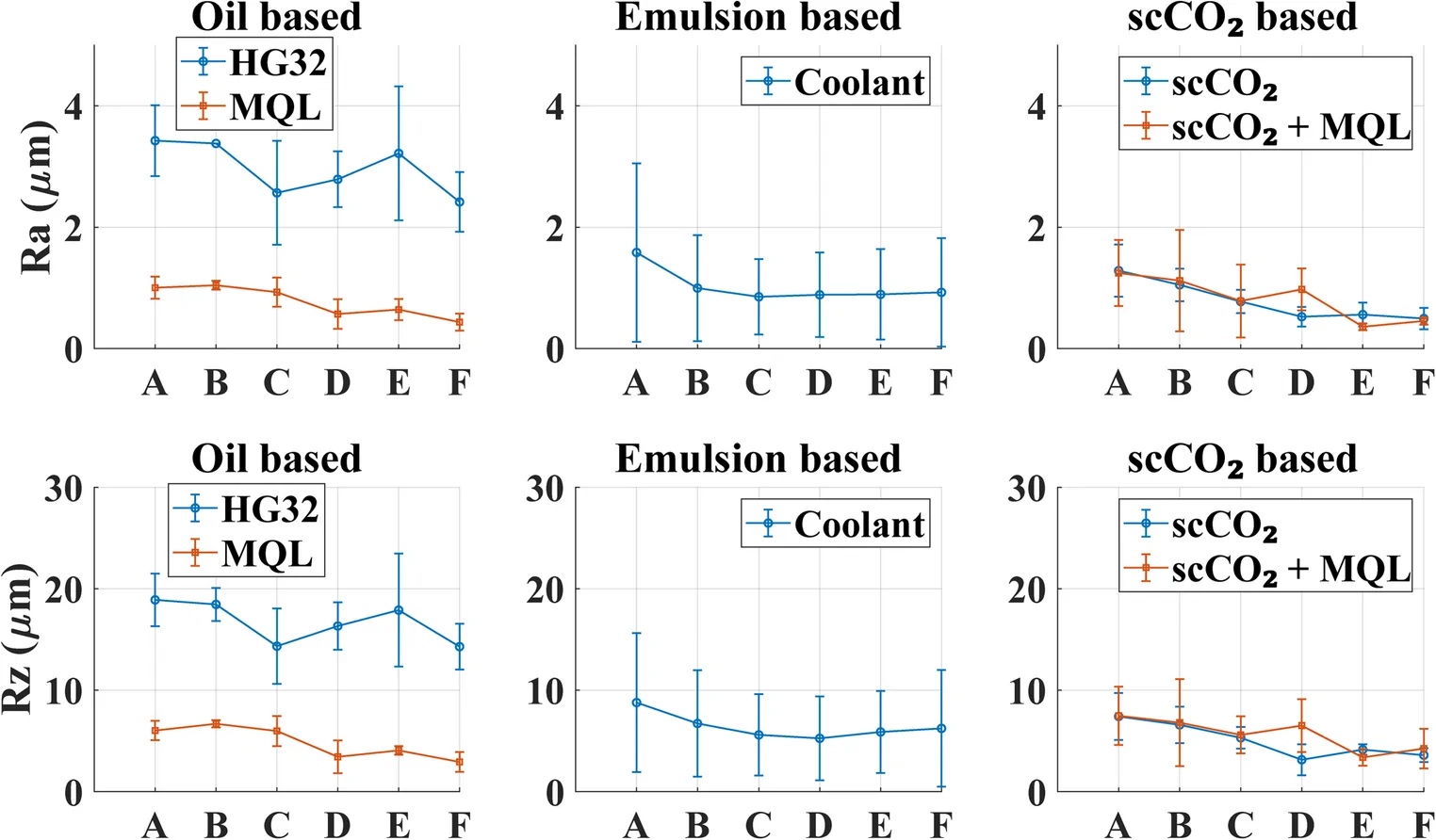
Ra and Rz plots for Feed 500 mm/min and Spindle speed 0 RPM

Surface quality was evaluated using the arithmetic mean roughness (Ra) and the maximum height of the profile (Rz), measured at the six predefined locations (A to F). For the condition Feed 500 mm/min, Spindle speed 0 RPM, three repeated tests were performed for each lubrication strategy, and the results are presented as mean values with error bars representing the standard deviation. For the Feed 750 mm/min, Spindle speed 50 RPM condition, only a single repeat was available for each lubrication option due to material and time constraints, therefore, no statistical dispersion is shown for this condition. Figure 11 represents the Ra and Rz distributions along the formed part for all lubrication strategies at a feed rate of 500 mm/min and zero spindle rotation. Clear differences in surface roughness are observed between oil based, coolant based, and scCO₂ based lubrication strategies. Across all locations, MQL consistently produced the lowest Ra and Rz values, followed by scCO₂ and scCO₂ + MQL, while machine coolant and HG32 oil exhibited comparatively higher roughness levels. HG32 oil shows the highest Ra and Rz values at most locations, particularly along the sidewall region (A to C).

For all lubrication strategies, both Ra and Rz generally decrease from locations near the start of forming (A, D) toward the end of the forming depth (C, F). This trend is more pronounced for MQL and scCO₂ based strategies. Error bars indicate relatively lower spread for MQL, while larger scatter is observed in HG32 and emulsion coolant-based lubrication method, suggesting increased sensitivity to local contact conditions. Although HG32 oil resulted in the lowest forming forces, it produced comparatively the poorest surface quality. In contrast, MQL achieved the best surface finish despite generating higher forming forces than HG32. scCO₂ and scCO₂ + MQL show similar Ra and Rz magnitudes, with slightly improved consistency relative to coolant. The surface quality results highlight that low forming force does not directly translate to improved surface finish. The higher Ra and Rz values associated with HG32 oil are attributed to boundary dominated lubrication, where insufficient debris removal and local lubricant starvation promote adhesive transfer and micro ploughing at the tool sheet interface. MQL provides superior surface quality due to the formation of a stable lubricating film combined with pressurised air, which promotes continuous removal of microscopic wear debris and limits adhesion. This results in smoother sliding conditions supported with lower friction indicator values despite moderate force levels. scCO₂ and scCO₂ + MQL strategies, while producing higher forces due to their cooling-dominated behaviour, offer improved surface consistency compared to HG32 by reducing adhesive wear and suppressing galling. However, under zero spindle rotation conditions, the strong cooling effect limits localised thermal stabilisation, preventing further improvements in surface roughness relative to MQL. Machine coolant primarily acts as a cooling and flushing medium, reducing adhesion but lacking sufficient boundary lubrication, which explains the moderately higher Ra and Rz values.

**Fig. 12:**
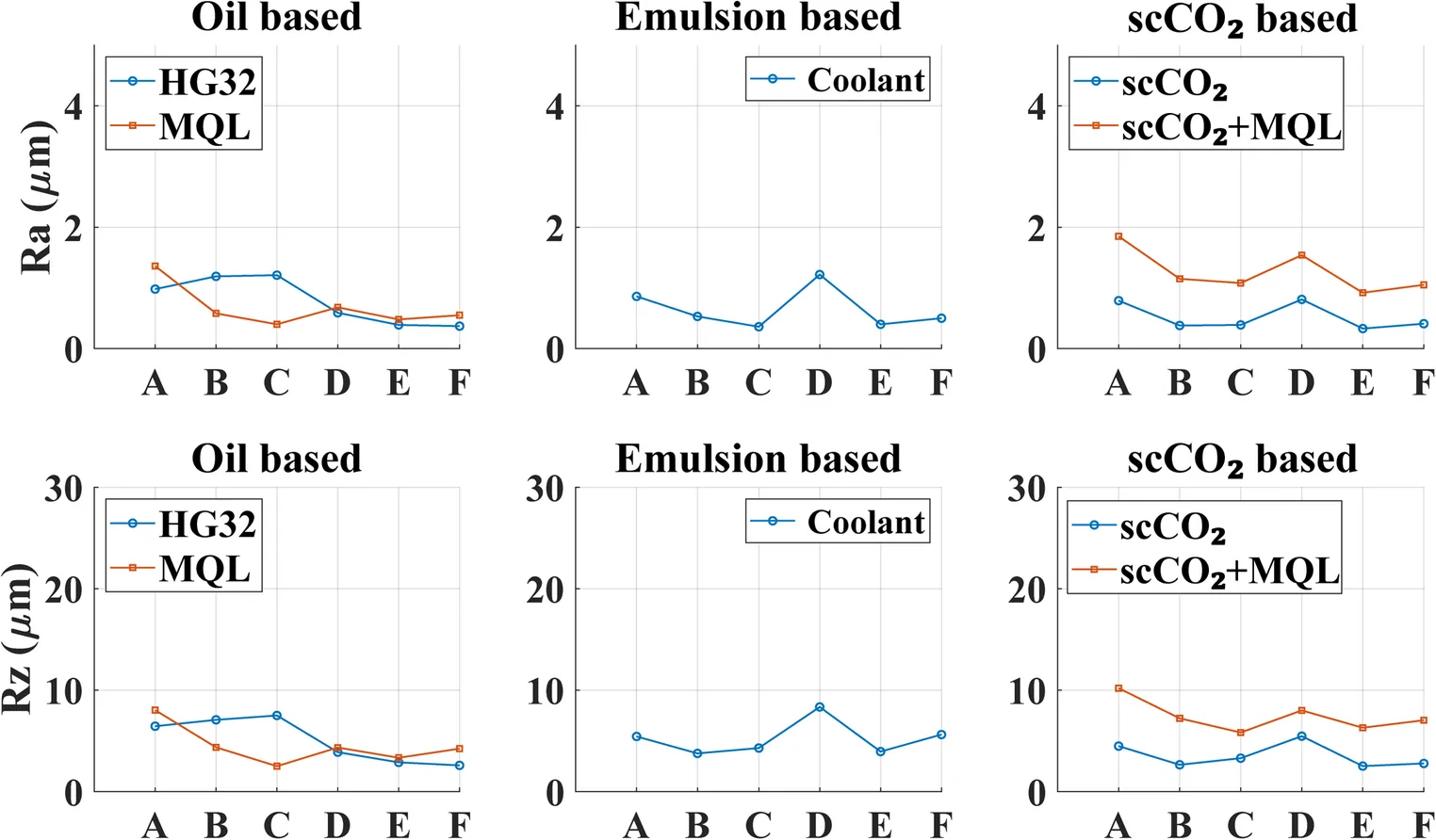
Ra and Rz plots for Feed 750 mm/min and Spindle speed 50 RPM

Figure 12 presents Ra and Rz measurements at a feed rate of 750 mm/min with a spindle speed of 50 RPM. It should be noted that, for the condition feed rate 750 mm/min and spindle speed 50 RPM, only a single formed component was available for each lubrication strategy due to material availability and experimental time constraints. Consequently, statistical dispersion between repeated forming trials were not evaluated for this condition. However, the reliability of the reported surface roughness values is supported by the dedicated Alicona measurement repeatability study presented in Sect. 4.2.1, in which three independent repeated surface scans were performed at all six measurement locations and demonstrated a low standard deviation relative to the corresponding mean Ra and Rz values, confirming high measurement stability and repeatability of the optical surface characterisation method. Therefore, although part-to-part repeatability could not be assessed for the 750 mm/min and spindle speed 50 RPM condition, the observed trends in Fig. 10 are considered representative and not influenced by measurement uncertainty, allowing a meaningful qualitative and comparative assessment of lubrication effects under this higher feed and rotational speed forming regime. Overall surface roughness values are reduced compared to the non-rotation condition, especially for the MQL lubricant. MQL again produces the lowest Ra and Rz across the locations, followed by HG32 and scCO₂ based strategies. Emulsion coolant exhibits moderate roughness levels, with noticeable variation along the forming depth (i.e. location C to location D).

With the introduction of spindle rotation (feed rate 750 mm/min and spindle speed 50 RPM), the Ra and Rz values become more uniform across all measurement locations, indicating an overall improvement in surface consistency compared with the non-rotating condition. Local roughness peaks observed under zero spindle rotation are notably reduced, particularly for the scCO₂ and emulsion coolant-based strategies. The diagonal edge and planar wall regions exhibit comparable roughness levels, suggesting a more homogeneous contact condition during forming. HG32 shows a clear improvement in Ra and Rz compared with the non-rotating case, however, its surface quality remains inferior to that obtained with MQL. scCO₂ and scCO₂ + MQL produce similar surface roughness levels, with no consistent advantage observed for the hybrid approach under the present conditions. MQL remains the most effective lubrication strategy, providing the lowest and most stable Ra and Rz values due to its balanced lubrication capability, effective debris removal, and controlled heat retention. Although scCO₂ based strategies continue to exhibit higher forming forces, their surface quality benefits are primarily associated with reduced adhesive wear and galling under rotating conditions, rather than enhanced material softening or improved material flow.

#### Surface texture plots - (feed rate 500 & 750 mm/min, spindle speed 0 & 50 RPM)

Surface texture plots provide a qualitative representation of the topographical features generated during incremental sheet forming, complementing the friction indicator evolution value and quantitative roughness metrices (Ra and Rz). Unlike scalar parameters, texture maps reveal the orientation, continuity, and severity of surface features such as tool marks, material smearing, adhesive patches, and micro-ploughing tracks. These plots are useful for identifying dominant tribological mechanisms and for visually correlating surface damage with lubrication strategy and process conditions.

**Fig. 13:**
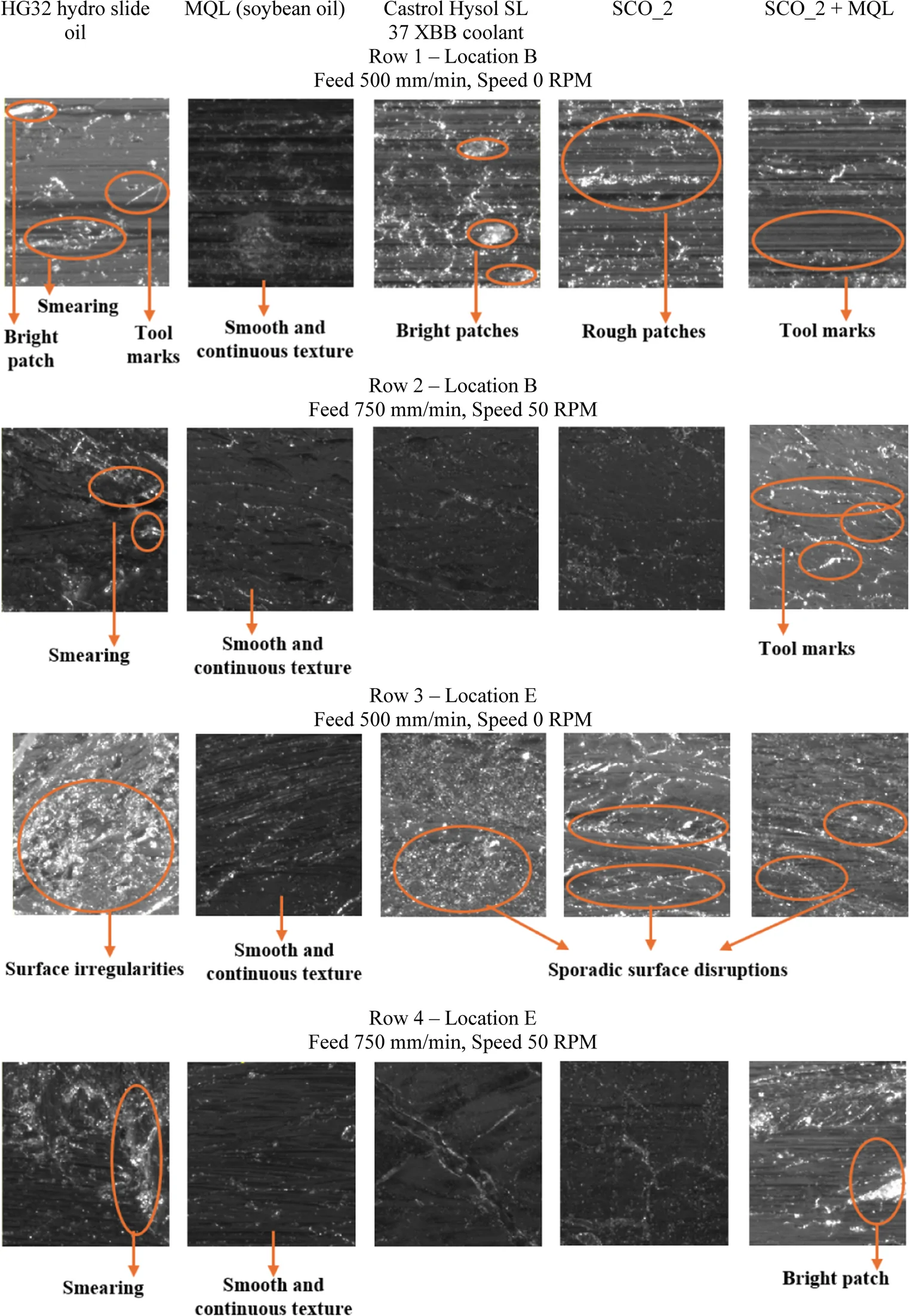
Texture plots – Location B and E

In Fig. 13, the surface texture plots are grouped by measurement location. Location B (centre of the side wall) is shown together for both process conditions (rows 1 and 2), while location E (centre of diagonal edge) is shown together for both conditions (rows 3 and 4). Location B represents a region dominated primarily by plane-strain deformation, whereas location E is subjected to combined deformation modes arising from the convergence of two wall directions and is therefore more sensitive to lubrication effects.

For location B under the non-rotation condition (Feed 500 mm/min, Spindle speed 0 RPM – row 1), HG32 hydro-slide oil produces pronounced directional tool marks, extensive material smearing and local bright patches associated with adhesive transfer, consistent with the high Ra and Rz values reported in Sect. 4.2.2 and indicative of boundary-dominated lubrication with limited debris removal. In contrast, the MQL (soybean oil) condition exhibits the smoothest and most uniform texture, with fine and continuous tool marks and minimal evidence of adhesion or surface tearing. The emulsion coolant condition shows interrupted tool marks and localised rough regions. The scCO₂ and scCO₂ + MQL strategies generate relatively uniform tool mark patterns with reduced adhesive features compared to HG32, but the surface remains slightly rougher than under MQL, reflecting the cooling-dominated nature of scCO₂ lubrication in the absence of spindle rotation.

**Fig. 14:**
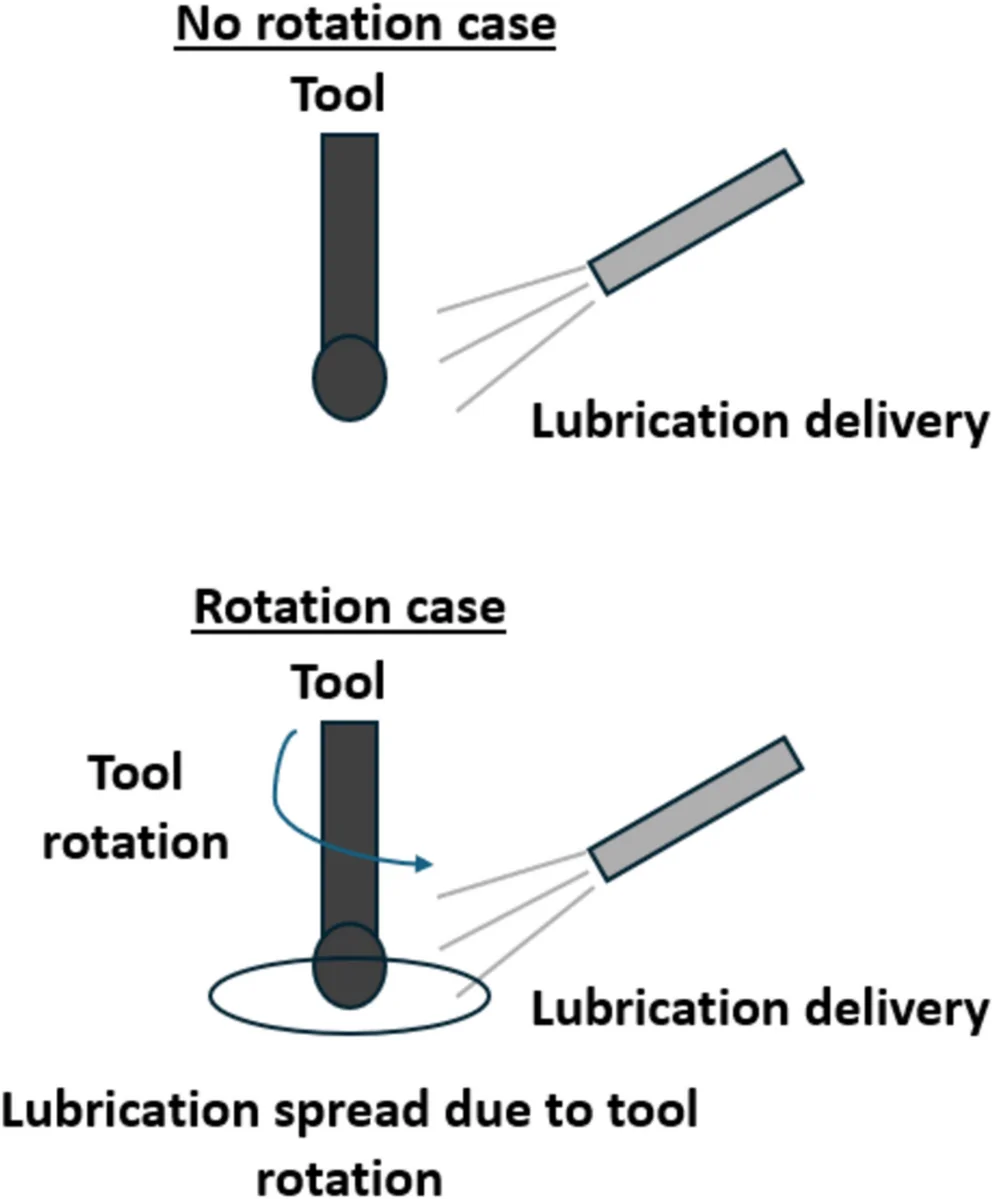
Lubrication spread visualisation

For location B under the rotating condition (Feed 750 mm/min, Spindle speed 50 RPM – row 2), all lubrication strategies exhibit smoother and more homogenous textures than in the non-rotating case. With spindle rotation, the lubricant is likely to distribute more effectively across the tool-sheet contact surface, whereas without rotation the lubricant spread at the tool-sheet interface is more limited (Fig. 14). MQL again produces the most uniform texture, characterised by fine, well aligned tool marks. HG32 shows a clear improvement relative to the non-rotating case, with reduced smearing and fewer adhesion features, although its tool marks remain more pronounced than those observed under MQL. Both scCO₂ based strategies display reduced adhesion and more regular tool mark patterns at location B.

For location E (Rows 3 to 4), which corresponds to the diagonal edge region, the surfaces exhibit generally higher sensitivity to lubrication strategy. Under the non-rotating condition (row 3), HG32 produces sever smearing and irregular surface features, while MQL maintains comparatively smooth and continuous textures. Emulsion coolant and scCO₂ based strategies show sporadic surface disruption and locally intensified tool marks, reflecting the combined deformation state and reduced stability of the lubrication film in this region. Under the rotating condition (row 4), surface textures at location E become more uniform for all strategies, however, subtle surface disruptions remain visible, particularly for the emulsion coolant and scCO₂ based lubrication, indicating the increased tribological demand along the diagonal edge.

While HG32 consistently minimises forming force, it promotes adhesive wear and surface smearing leading to inferior surface texture. In contrast, MQL provides the most favourable balance between lubrication, debris removal, and controlled heat retention, resulting in superior surface quality at both locations and under both process conditions. scCO₂ based strategies, although associated with higher forming forces due to their cooling-dominated behaviour, effectively suppress galling and large-scale adhesion, particularly when combined with spindle rotation. This highlights the trade-off between tool loading and surface quality in ISF and demonstrates the value of surface texture analysis in revealing lubrication driven tribological behaviour beyond conventional roughness metrics.

### Geometrical deviations

Geometrical deviation analysis was performed to quantify the dimensional accuracy and shape fidelity of the formed pyramids under different lubrication strategies. ISF is well known to introduce geometric errors due to elastic recovery, sheet bending, localised thinning, and non-uniform material flow, which lead to deviations from the nominal CAD geometry. In the present study, full field deviation was evaluated by comparing the scanned “as formed” surface to the nominal CAD model, allowing the influence of lubrication strategy on global and local accuracy to be assessed. The deviation distributions are presented as (a) full field contour plots showing the overall deviation pattern across the part, and (b) and (c), diagonal centre-line deviation profiles extracted along the 45° and 135° diagonals, providing higher spatial resolution from edge to edge through the part centre. Positive deviations indicate outward displacement relative to the CAD model, whereas negative deviations indicate inward displacement. For both process conditions, a consistent deviation scale was used to enable direct visual comparison between lubrication strategies.

#### Geometrical deviations – feed 500 mm/min, spindle speed 0 RPM

**Fig. 15:**
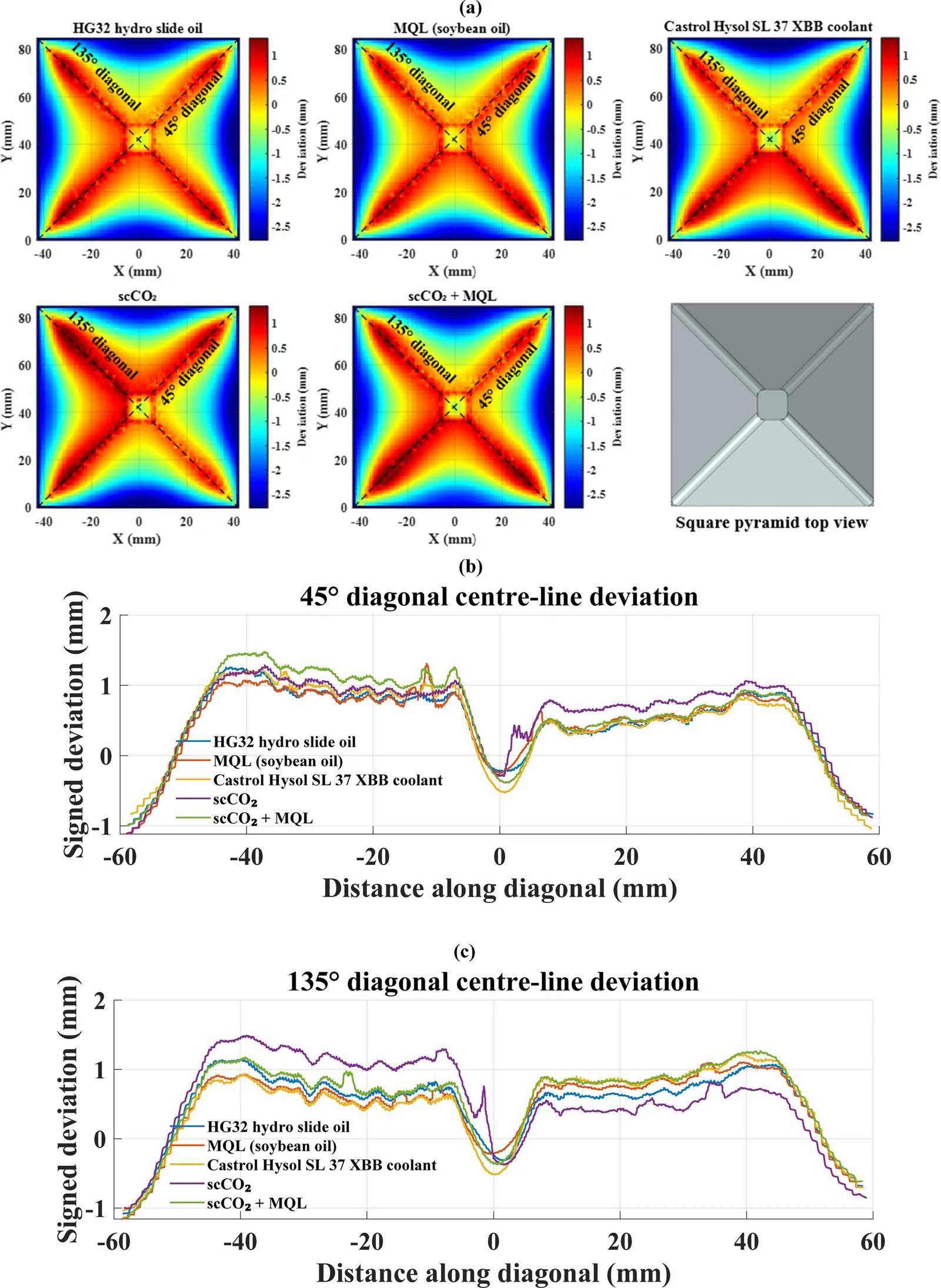
Geometrical deviation contour plot with 45° and 135° – Feed 500 mm/min, Spindle speed 0 RPM: (**a**) Full field contour plot, (**b**) diagonal centre line deviation profile along 45°, (**c**) diagonal centre line deviation profile along 135°

Figure 15a shows the deviation contour maps and diagonal line profiles for the Feed 500 mm/min, Spindle speed 0 RPM condition. All lubrication strategies exhibit a similar global deviation pattern characterised by a pronounced “X-shaped” deviation distribution, with the largest deviations aligned along the diagonal directions and reduced deviations near the mid wall regions. This deformation morphology is typical of square pyramid ISF parts and reflects the interaction between toolpath progression, elastic recovery, and directional stiffness variations across the formed sheet. The 45° and 135° diagonal line plots (Fig. 15b and c) show that the deviation magnitude varies significantly along the diagonal length, with peak positive deviations occurring away from the centre and sharp deviation transitions near the part centre. While the overall trend is consistent for all lubrication options, the magnitude and asymmetry of deviation differ between lubrication strategies. For most lubrication methods, deviations increase progressively from the outer edges toward the diagonal mid length region, followed by a strong drop near the centre (around 0 mm along the diagonal), and then rise again towards the opposite side. This indicates that the part does not deform uniformly and that the central region behaves as a transition zone due to the interaction of local bending and spring back. scCO₂ based strategies show increased scatter and more pronounced separation between the two diagonals, indicating a stronger asymmetry and higher sensitivity to local forming conditions when compared to oil based and coolant-based strategies. The hybrid scCO₂ + MQL condition shows the largest deviation excursions along portions of the 45° diagonal.

A key observation is that, at the Feed 500 mm/min, Spindle speed 0 RPM condition, lubrication strategies that produced higher forming forces (scCO₂ and scCO₂ + MQL) also tend to show larger geometric deviations and stronger asymmetry in the diagonal profiles. In contrast, the oil-based strategies (HG32 and MQL), which reduced forming forces, exhibit smoother deviation trends and slightly improved geometric stability. Machine coolant shows consistent deviation profiles, but with deviation magnitudes similar to the oil-based cases. Lubricants that promote heat retention and reduce friction (particularly MQL and HG32) are likely to enable smoother material flow and reduced elastic recovery gradients, leading to comparatively more stable deviation profiles. In contrast scCO₂ based strategies impose strong recoiling at the interface which increases sheet flow stress and raises forming forces. Higher forces increase the elastic energy stored in the sheet and clamping system, which can increase spring back during unloading and lead to larger geometrical deviations. The increase deviation asymmetry observed for the scCO₂ and scCO₂ + MQL cases also suggest that cooling dominated lubrication may amplify the local stiffness imbalance or contact instability during toolpath progression.

#### Geometrical deviations – feed 750 mm/min, spindle speed 50 RPM

**Fig. 16:**
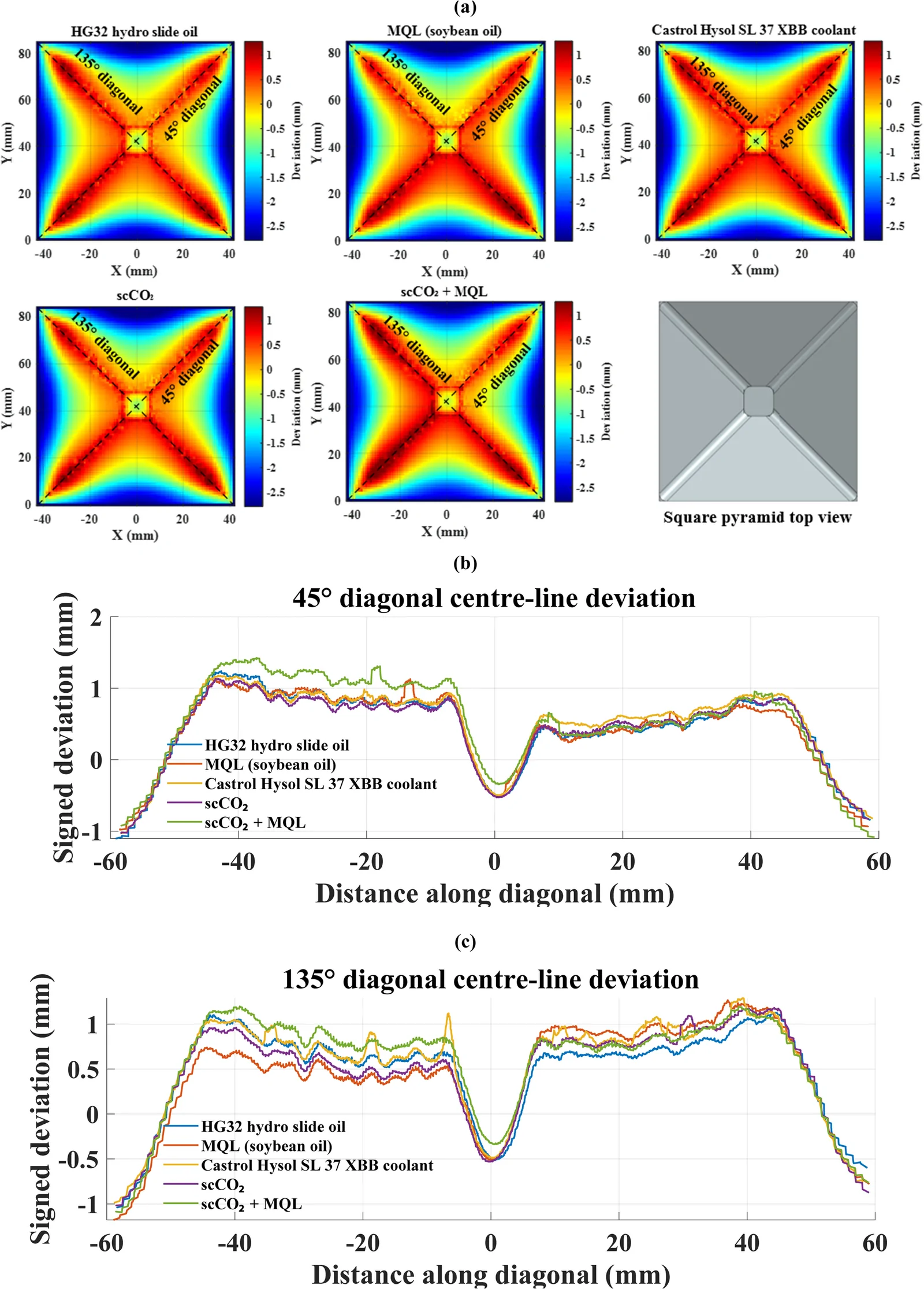
Geometrical deviation contour plot with 45° and 135° – Feed: 750 mm/min, Speed 50 RPM: (**a**) Full field contour plot, (**b**) diagonal centre line deviation profile along 45°, (**c**) diagonal centre line deviation profile along 135°

Figure 16a presents the deviation contour maps and diagonal line profiles for the Feed 750 mm/min, Spindle speed 50 RPM condition. Similar to the Feed 500 mm/min, Spindle speed 0 RPM condition, the deviation contour plots show an “X-shaped” deviation pattern for all lubrication strategies, indicating that the global deformation morphology remains consistent. However, the overall deviation range is reduced, and the diagonal deviation profiles are smoother, indicating improved shape stability under this process condition. The diagonal plots (Fig. 16b and c) reveal reduced separation between lubrication strategies compared to the non-rotation condition, with closer clustering of the deviation curves in both 45° and 135° directions.

Across all lubrication strategies, deviations evolve more gradually along the diagonal length compared with Feed 500 mm/min, Spindle speed 0 RPM condition. The sudden deviation transition near the centre remains present but is less severe. The diagonal deviation curves for oil based and coolant-based lubrication show improved overlap, while scCO₂ based strategies remain slightly more dimensionally deviated from the group in selected regions. Compared with the non-rotation case, the introduction of spindle rotation reduces the deviation difference between lubrications. MQL and HG32 show consistent geometric responses and relatively stable diagonal profiles. Coolant produces comparable shape accuracy, with only localised deviations visible. scCO₂ and scCO₂ + MQL still show slightly larger deviation excursions in some regions, but the difference is notably smaller than under the Feed 500 mm/min, Spindle speed 0 RPM condition. These results indicate that the interaction between lubrication strategy and spindle rotation plays a key role in defining both forming mechanisms and geometric accuracy. From a forming perspective, the results highlight a trade-off: lubrication strategies that enhance cooling may improve surface quality, but they can also increase forming forces and geometric deviation under conditions where heat retention is limited.

Across both forming conditions, the global deviation morphology remains consistent, with diagonal dominated deviation patterns typical of pyramidal ISF components. However, lubrication strategies influence deviation magnitude and symmetry, particularly under non-rotating conditions. Oil based lubrication, especially MQL, provides the most consistent deviation profiles, while scCO₂ based strategies show increased deviation magnitudes and asymmetry at the Feed 500 mm/min, Spindle speed 0 RPM condition. Introducing spindle rotation reduces these differences and improves geometric stability for all lubrication strategies.

## Conclusions

This study investigates the influence of diverse lubrication strategies (HG32 hydro slide oil, flood emulsion coolant, MQL soybean oil, supercritical carbon dioxide (scCO₂), and scCO₂ + MQL) on incremental sheet forming (ISF) of 1 mm thick stainless-steel 304 into a square pyramid geometry. Forming force evolution, surface quality (Ra, Rz), and full field geometric accuracy were evaluated under two process conditions: (1) Feed 500 mm/min, Speed 0 RPM and (2) Feed 750 mm/min, Speed 50 RPM, supported by repeatability trials and tolerance-based deviation mapping. The main conclusions are as follows.

- Lubrication strategy strongly affects forming mechanisms and part quality, influencing forming forces, friction indicator evolution, surface roughness, and dimensional accuracy through changes in process parameters.
- HG32 consistently produces the lowest vertical forming forces. However, HG32 also generated a higher friction indicator evolution trend, the poorest surface quality, with higher Ra and Rz values and visible evidence of smearing/adhesion, indicating that low force does not necessarily translate to improved surface quality.
- MQL soybean oil achieved the best overall surface quality, having a lower and the most stable friction indicator evolution trend (an indication of lubrication efficiency), producing the lowest Ra and Rz across the measured locations. Despite generating higher forces than HG32, MQL provided the most stable lubrication condition and reduced surface damage considered to be via improved film formation and debris evacuation.
- Emulsion coolant produced intermediate performance, offering effective cooling and flushing, but with less consistent surface quality than MQL, particularly in Rz indicating occasional localised surface disruption due to limited boundary lubrication strength.
- scCO₂ and scCO₂ + MQL generated the highest forming forces, especially under Feed 500 mm/min and Speed 0 RPM conditions, consistent with a cooling-dominated contact condition that increases flow stress and force demand. Although force levels were higher, scCO₂ based strategies generally reduce severe adhesion effects relative to HG32 and produced moderate surface quality.
- Geometrical deviation trends were broadly similar across lubrications, showing the typical diagonal dominated deformation pattern of ISF square pyramids. However, MQL provided the most consistent geometric accuracy, while scCO₂ based strategies tended to show increased deviation and asymmetry at Feed 500 mm/min and Speed 0 RPM.
- The Feed 750 mm/min, Speed 50 RPM condition produced the most favourable combined outcomes, with generally improved surface finish and reduced geometric deviation across all lubrications. The introduction of spindle rotation improved contact stability and reduced the separation between lubrication strategies’ performance.
- Increasing feed rate from 500 to 750 mm/min reduced cycle time without compromising part quality, indicating that productivity can be improved while maintaining acceptable surface quality and geometric performance, particularly when combined with appropriate lubrication.

Overall, the results show that MQL-delivered soybean oil offered the best balance of surface quality and dimensional accuracy whilst being highly economical, environmentally friendly and easy to implement in industrial applications. Meanwhile HG32 minimised force but compromised surface quality, and scCO₂ based strategies increased force due to cooling effects but may support improved surface quality through reduced adhesion mechanisms. The findings provide a clear process performance dataset for selecting lubrication strategists in ISF of hard to form stainless-steel components.

## Data Availability

The data supporting the findings of this study are available from the corresponding author upon reasonable request, subject to institutional approval.

## Abbreviations

CAD: Computer—Aided Design
CNC: Computer Numerical Control
CO₂: Carbon Dioxide
F500, S0: Feed Rate 500 mm/min, Spindle Speed 0 RPM
F750, S50: Feed Rate 750 mm/min, Spindle Speed 50 RPM
HG32: Hydro—Slide Oil (ISO VG 32 class)
ICP: Iterative Closest Point
IFDR: Intelligent Fluid Delivery & Recovery
ISF: Incremental Sheet Forming
kNN: k—Nearest Neighbour
MQL: Minimum Quantity Lubrication
PCA: Principal Component Analysis
Ra: Arithmetic Mean Roughness
RSm: Mean Spacing of Profile Elements
Rz: Maximum Height of Profile
µ*: Friction Indicator
scCO₂: Supercritical CO₂
SPIF: Single Point Incremental Forming
STL: Standard Tessellation Language (mesh format)
